# Burrowing behavior is a potential non-invasive proxy for lesion development in a syngeneic murine model of endometriosis

**DOI:** 10.1186/s12905-025-04112-4

**Published:** 2025-12-12

**Authors:** Megha Anchan, Samruddhi Deshpande, Atharvaraj Hande, Rooma Sinha, Jayesh Mudgal, K. Nandakumar, Rahul Dutta

**Affiliations:** 1 Division of Reproductive Biology, Department of Reproductive Science, Kasturba Medical College, Manipal, Manipal Academy of Higher Education, Manipal, Karnataka 576104 India; 2 Department of Gynecology, Apollo Health City, Hyderabad, Telangana India; 3 Department of Pharmacology, Manipal College of Pharmaceutical Sciences Manipal, Manipal Academy of Higher Education, Manipal, Karnataka 576104 India; 4 Center for Animal Research, Ethics & Training, Manipal Academy of Higher Education, Manipal, Karnataka 576104 India

**Keywords:** BALB/c, C57BL/6j, Swiss albino, Burrowing assay, Endometriosis incidence

## Abstract

**Background:**

Endometriosis (EM) is a persistent, chronic inflammatory condition associated with excruciating pelvic pain and infertility. The absence of a pre-clinical model that reliably replicates the clinical and functional hallmarks of human EM continues to limit progress in the domain. Furthermore, no rodent model developed to date has achieved a 100% incidence rate, compromising the reproducibility of existing models. Further, the inability to detect lesion development without sacrificing the animal presents a significant barrier for preclinical interventional trials designed to improve the management of EM.

**Methods:**

We employed a non-invasive method based on the altered burrowing behavior of the animal to predict lesion development in the syngeneic mice EM model. We used the burrowing assay (BA) before dissection as a non-invasive behavioral marker to evaluate lesion progression across three distinct laboratory strains: C57BL/6j, BALB/c, and Swiss albino to account for variation due to genetic, immunological, and strain specificity.

**Results:**

EM mice displayed a significant decline in burrowing activity compared to controls across all three strains. Based on BA performance, recipient mice were stratified into two groups: Recipients with a low burrow score (LB) and those with a high burrow score (HB). Additionally, LB mice exhibited decreased exploratory behavior and increased sensitivity to thermal pain. In contrast, HB mice had exploratory and thermal responses comparable to those of the control group. Post-dissection, LB mice were presented with ectopic lesions (LB+), whereas HB mice were lesion-negative (HB-). BA performance correlated strongly with lesion presence via ROC analysis, with a combined AUC of 0.883 (and an AUC of 1 for C57BL/6j), indicating excellent diagnostic accuracy of BA in predicting EM incidence. The combined approach of correlating burrowing behavior with other evoked behavioral responses and non-evoked provided a comprehensive assessment of EM disease progression.

**Conclusion:**

To our knowledge, our research provides evidence for the feasibility of ethologically valid burrowing behaviour as a non-invasive predictor of EM incidence in the syngeneic mice model. Future preclinical drug research for EM management could leverage BA to identify and select only lesion-positive animals for intervention trials. This approach will enhance the translational value of EM research.

**Supplementary Information:**

The online version contains supplementary material available at 10.1186/s12905-025-04112-4.

## Background

Endometriosis (EM) is one of the most prevalent reproductive disorders among women. It is characterized by the presence of viable endometrial tissue growing in extra-pelvic regions [1]. This gynecological disorder is prevalent in 10% of women of reproductive age [2], with increased frequencies in those experiencing dysmenorrhea/chronic pelvic pain (CPP) (40–60%), subfertility (30–50%), and pelvic discomfort (71–87%), which are the frequent manifestations of EM [3]. Despite its high prevalence and significant impact on quality of life, the variability in EM severity and lesion traits indicates a complex and not thoroughly understood etiology [4]. Murine models have been widely and effectively exploited to investigate EM. Among these, the syngeneic approach, which involves intraperitoneal (i.p) injection of mouse endometrial tissue into immune-compatible hosts, is extensively utilized for in vivo EM modelling. However, it is limited by an inconsistent lesion induction rate (70–80%) after injecting the donor endometria [5, 6]. Hence, without clear confirmation of lesion existence in all EM-induced animals, the experimental groups risk including lesion-negative animals before the intervention, potentially diluting the treatment effects. Thus, a partial success rate introduces a limitation for intervention research. Further confounding this uncertainty, the EM induction success rate is assessed only upon dissection [6–10]. The advanced imaging techniques, such as bioluminescent genetic manipulation, contrast agents, high-resolution ultrasound, and MRI, have been explored as non-invasive alternatives to monitor and track the course of EM lesions in real-time [11–18]. But these approaches are resource-intensive, technically demanding, and not routinely used in many research laboratories. Therefore, there is a pressing need for a simple, cost-effective, reliable approach to identify lesion-positive animals during the experimental timeline.

Burrowing is a conserved, spontaneous behavior in rodents that reflects an animal’s general well-being and motivation to interact with its environment [19]. Decreased burrowing has been observed in various models of prion disease [20], brain lesions [21], complex pain syndrome [22], LPS-induced systemic inflammation [23], Alzheimer’s disease [23], and post-laparotomy pain [24], making it a promising non-evoked behavioral readout. Recent investigations have also demonstrated low burrowing performance in a mouse model of EM [25], showing that lesion-induced pain may affect spontaneous behaviors. However, whether burrowing behavior can be employed as a credible predictor of lesion development and disease progression has not been investigated. In this study, we leveraged a basic experimental setup of BA [19], to evaluate alterations in “non-evoked pain” by monitoring the behavioural alterations of EM mice. Our study aimed to determine if this single assay could provide valuable information regarding lesion burden in a syngeneic EM mouse model.

## Methods

### Animal maintenance

Adult female C57BL/6j, BALB/c, and Swiss albino mice (8 weeks old, weighing 22–28 g) were procured from the Central Animal Research Facility at Manipal Academy of Higher Education. They were housed in standard conditions of controlled temperature (23 ± 2 °C), humidity (50–55%), and light-dark cycle (12–12 h) with *ad libitum* access to water and food throughout the study. Animals were group-housed (4–5 per cage) in ventilated polypropylene cages with husk bedding. The study was approved by the Institutional Animal Ethics Committee at Kasturba Medical College, Manipal (Approval Numbers: BALB/c- IAEC/KMC/45/2022, C57BL/6j- IAEC/KMC/88/2024, Swiss albino- IAEC/KMC/56/2022). The animal numbers were determined based on previous studies utilizing the syngeneic endometriosis mouse model [26]. Institutional guidelines and the guidelines of the Committee for the Purpose of Control and Supervision of Experiments on Animals (CPCSEA)were strictly followed for animal handling, and the reporting of animal experiments follows ARRIVE (Animal Research: Reporting of In vivo Experiments) guidelines.

### Experimental design and timeline

We planned a systematic study using three strains of commonly used laboratory mice, C57BL/6j, BALB/c, and Swiss albino. We employed a total of 57 C57BL/6j (18 controls, 13 donors, and 26 recipients), 73 BALB/c (25 controls, 16 donors, and 32 recipients), and 58 Swiss albino (16 controls, 14 donors, and 28 recipients) mice for EM induction. This number variation resulted from the logistics involved in a large-scale initial screening to ensure an adequate number of mice across different genetic backgrounds were included for the primary validation of the BA. A syngeneic EM mice model was developed by i.p injecting the donor uterine fragments (UF) into the abdominal cavity of recipient mice. BA was used as the first screening tool to obtain a burrowing score, and a total of *n* = 26 for C57BL/6j, *n* = 32 for BALB/c, and *n* = 28 for Swiss albino mice were used. Then, all the recipients were chosen for the subsequent behavioral assays. It comprised of, LB (1) OFT, C57BL/6j (*n* = 8 control and *n* = 16 LB), BALB/c and Swiss albino (*n* = 8 control and *n* = 8 LB), and (2) Hot plate, C57BL/6j (*n* = 12 control, *n* = 24 LB), BALB/c and Swiss albino (*n* = 12 control, *n* = 12 LB), HB (1) OFT, C57BL/6j (*n* = 8 control and *n* = 8 HB), BALB/c and Swiss albino (*n* = 8 control and *n* = 4 HB), and (2) Hot plate (C57BL/6j, *n* = 8 control, *n* = 8 HB) BALB/c and Swiss albino (*n* = 8 control, *n* = 4 HB).

We generated a ROC curve, and a cut-off value of the predictive burrow score for EM mice was calculated. The controls and EM mice were dissected after 12 days. Following euthanasia by cervical dislocation, first, peritoneal fluid (PF) was collected by irrigating the abdominal cavity with sterile 1x PBS and immediately processed for flow cytometry (FC). Then, the blood was collected by cardiac puncture, and the isolated serum was stored at −80 °C for analysis of inflammatory cytokines by ELISA. The peritoneal cavity was surgically opened, and suspected EM/EM-like lesions were carefully excised. Animals were grouped according to the presence or absence of ectopic lesions as evaluated by burrowing scores. That is, a low burrow score with confirmed lesions (LB+), and a high burrow score without any lesions (HB-). The obtained ectopic lesions were photographed for documentation. Subsequently, they were fixed in Bouin’s solution for 24 h and then transferred to 70% ethanol for histological and immunohistochemical (IHC) characterization.

### Development of a syngeneic EM mouse model

The approach followed a protocol of EM induction that we have previously established [26]. Briefly, the donors and recipient mice were allowed to acclimate for 5 days before the experimental procedure, during which the estrous cycle stages were monitored. Upon confirmation of the mice’s normal cycling, they were randomly allocated to either the control, donor, or EM group. All animals in a specific cage received UF injection simultaneously to prevent cross-exposure to olfactory cues among them. The donor animals were primed with estradiol benzoate (EB) (TCI chemicals, #E0329) subcutaneously (s.c.) at a dose of 3ug/mouse for 7 days to stimulate the growth of endometrium. Then, the donor mice were sacrificed, uterine horns (UH) were extracted, washed with sterile 1x Phosphate Buffer Saline (1x PBS) containing penicillin (100 U/mL) and streptomycin (100 mg/mL) (Pen/strep, Thermo Fisher Scientific #15140122), split longitudinally and minced into small cell aggregation suspension of UF (< 0.1 mm) consisted of both eutopic endometrium and uterine muscle. They were then reconstituted in 0.5 mL of 1x PBS without the pen/strep, passed through an 18-gauge needle (Dispovan #45678) to ensure the consistent size of the UF (approximately 1 mm), and then i.p injected into 2 recipient mice. By this process, each mouse receives endometrium originating from half a uterus. Mice in the control group were injected with EB (s.c.) and 1x PBS (i.p.) at the same dose and time points. To limit fluctuations and maintain steady levels of estrogen, the recipient animals were primed with a single dose of EB before the UF injection to synchronize the estrous cycle. All recipients received a dose of EB every 3 days until sacrifice to maintain estrogen in circulation.

### Assessment of anxiety in EM mice using behavioral studies

**Fig. 1:**
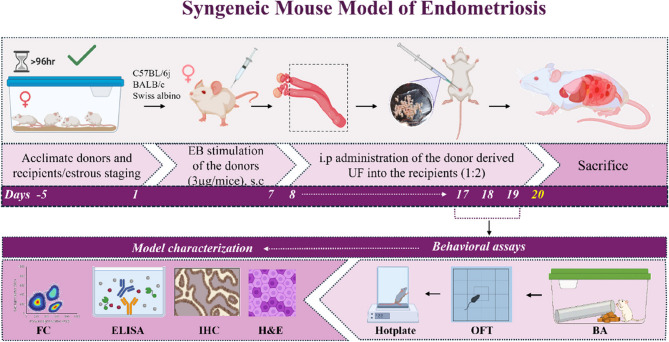
Experimental timeline and the workflow for the development of a syngeneic mouse model of EM: EM was induced in three strains of mice (C57BL/6j, BALB/c, and Swiss albino) via i.p. injection of donor UF. Adult female donor and recipient mice were acclimated for ≥ 96 h (Day 5), followed by s.c. injection of EB (3 µg/mouse) to stimulate endometrial proliferation in donors (day 1) for 7 days. On day 8, uteri were harvested, and UF was i.p injected into the recipient mice to induce ectopic lesion formation. On days 17 to 19, the behavioural assessment studies, such as BA for non-evoked behavioral assessment and spontaneous behavior, OFT to evaluate exploratory and anxiety-related behavior, and hot plate test (thermal sensitivity) to assess thermal nociception, were performed. All the recipients were sacrificed along with the control mice at day 20 for endpoint analysis. The EM model validation included FC analysis of peritoneal immune cells, ELISA of serum estrogen levels, H&E, and IHC to characterize the ectopic lesions. (Created in part with BioRender.com). (s.c- Subcutaneous, i.p- Intraperitoneal, BA- Burrowing assay, OFT-Open field test, H&E- Hematoxylin and Eosin, IHC- Immunohistochemistry, ELISA- Enzyme Linked Immunosorbent Assay, FC-Flow cytometry)

Behavioral assays provide vital insights into the presentation of anxiety/pain in animal models of EM. To complement the BA, both evoked and non-evoked behavioral assessments were performed. It comprises OFT for spontaneous behavior and the hot plate test for thermal nociception. These two behavioral assays were performed sequentially on mice with low burrow scores in the order shown in Fig. 1. All behavioral assessments described herein, excluding burrowing, were performed between 09:00 and 16:00 h. BA was carried out between 16:00 and 18:00 h (12 h light (06:00–18:00 h)/dark cycle (18:00–09:00 h). All behavioral testing was performed by an investigator who was blinded to the experimental groups. To ensure uniformity and prevent bias, the same investigator carried out all measurements in a blinded approach. After testing, mice were returned to their original home cage.

#### BA

We adopted an assay standardised by Deacon with minor modifications [19]. To brief, both the controls and EM recipient mice were housed individually in separate cages. A burrow tube filled with 200 g of chow diet was placed against the wall of a cage with a layer of husk bedding. The water was supplied with no additional feed in the cage hopper for the mice, as it could distract them. Considering Deacon’s demonstration that the healthy mice increased their burrowing activity at the second trial and thereafter continued their high level of burrowing, the animals were given time to acclimate to the burrow setup [19]. The animals were first acclimated testing environment and the presence of an empty burrowing tube for 30 min to diminish novelty-related exploration. After habituation, a filled burrow tube holding 200 g of chow pellets was placed into the same home cage. We started the test three hours before the dark cycle to reduce circadian variability, as per previous studies [19]. The amount of pellets displaced (g) was assessed at two timepoints: 2 h (short-term) and after 16 h (overnight). The measurements were obtained during a singular, continuous testing phase, with no repeated trials executed. After the experiment, all the animals were returned to their respective original cages.

#### Validation of the established EM model by ROC curve analysis

We performed an ROC curve analysis to evaluate the diagnostic reliability of the burrowing pattern for EM incidence. The ROC analysis used the overnight burrow scores. The analysis compared the binary outcome of Lesion Presence (State 1: LB mice) versus Lesion Absence (State 0). State 0 was defined as the combined group of recipient mice that did not develop ectopic lesions (i.e., HB mice, or ‘No lesion’ mice) and the healthy Control mice. The overnight burrow scores of the control and EM mice were compared with the presence or absence of ectopic lesions via Pearson’s correlation coefficient (Table 1). The AUC from the plot was calculated to evaluate the sensitivity, specificity, cut-off points, and overall model quality, with statistical analysis conducted using the latest version, SPSS 30.0.0.0. The cut-off burrow scores for each strain and the combined cohort were indeed determined by maximizing the Youden’s Index. The Youden’s Index is calculated as: Youden’s Index = Sensitivity + Specificity − 1. This index identifies the optimal cut-off value that maximizes the sum of sensitivity (true positive rate) and specificity (true negative rate) for the binary outcome.

**Table:** 

State	Definition in ROC Analysis
State 1 (Positive)	Recipient mice that developed ectopic lesions (post-dissection confirmation).
State 0 (Negative)	Recipient mice that did not develop ectopic lesions (post-dissection confirmation, High Burrow (HB) mice) and the Control mice.

**Table 1:** Burrow score from EM mice with lesion presence or absence used for generating ROC

Strain	Status	Burrow Score (g)	Strain	Status	Burrow Score (g)	Strain	Status	Burrow Score (g)
C57BL/6j	Lesion(1)	1	BALB/c	Lesion(1)	36.12	Swiss albino	Lesion(1)	0
(*n* = 20)	Lesion(1)	1	(*n* = 32)	Lesion(1)	36.12	(*n* = 28)	Lesion(1)	0
	Lesion(1)	1		Lesion(1)	38.41		Lesion(1)	1.68
	Lesion(1)	3.45		Lesion(1)	38.41		Lesion(1)	2.63
	Lesion(1)	6.72		Lesion(1)	42.64		Lesion(1)	4.86
	Lesion(1)	7.58		Lesion(1)	42.64		Lesion(1)	14.06
	Lesion(1)	8.2		Lesion(1)	49.35		Lesion(1)	14.06
	Lesion(1)	10.6		Lesion(1)	49.35		Lesion(1)	14.78
	Lesion(1)	10.75		Lesion(1)	49.69		Lesion(1)	18.04
	Lesion(1)	15.6		Lesion(1)	49.69		Lesion(1)	19.89
	Lesion(1)	16.79		Lesion(1)	52.61		Lesion(1)	22.28
	Lesion(1)	25.4		Lesion(1)	70.2		Lesion(1)	22.61
	Lesion(1)	46		Lesion(1)	77.29		Lesion(1)	24.33
	Lesion(1)	113.6		Lesion(1)	77.42		Lesion(1)	24.33
	No Lesion(0)	121		Lesion(1)	94		Lesion(1)	25.18
	No Lesion(0)	125.4		No Lesion(0)	41.25		Lesion(1)	26.79
	No Lesion(0)	145.4		No Lesion(0)	70.29		Lesion(1)	26.79
	No Lesion(0)	154.3		No Lesion(0)	102.76		Lesion(1)	55.71
	No Lesion(0)	187.11		No Lesion(0)	151.4		Lesion(1)	84.3
	No Lesion(0)	194.67		No Lesion(0)	199		No Lesion(0)	77.29
							No Lesion(0)	87.62
							No Lesion(0)	101.86
							No Lesion(0)	101.86
							No Lesion(0)	115.2
							No Lesion(0)	121.63
							No Lesion(0)	121.65
							No Lesion(0)	145.86
							No Lesion(0)	200

#### Assessment of spontaneous animal behaviour by OFT

To evaluate whether the development of EM alters non-evoked behavioral patterns, we recorded the spontaneous behavior of both control and EM mice in the OFT [27]. The control and EM mice with low burrow score (LB) and high burrow score (HB) were placed individually into the center of an open Plexiglas box with a clear floor without the husk bedding (50 cm × 50 cm × 40 cm) in a highly illuminated room. The box is virtually marked into the center zone and the peripheral zones. The experimental mouse was placed in one corner of the box and permitted to explore for 15 min. During a 15-minute session, animals were evaluated on the number of central and peripheral entries and the time spent in the central and peripheral zones, total distance travelled, mean speed, freezing episodes and time, and total mobile and immobile time of the animals were evaluated using a video-tracking system (Logitech webcam C930e). The data was analyzed via ANY-maze 64-bit version 7.48 software (Stoelting, Wood Dale, IL). Mice with higher levels of anxiety tend to spend more time on the periphery and less time in the centre area. The total distance travelled and the mean speed were regarded as measures of locomotor activity.

#### Assessment of thermal hyperalgesia by the hot plate test

The hot plate test was carried out by using a commercially available Hot/Cold Plate instrument (Model 35100, Ugo Basile) and performed as described previously [27]. The temperature-regulated plate consisted of a metal plate surface, which was heated to a constant temperature of 55.0 °C ± 0.5 °C for heat stimuli. Mice were allowed to acclimate to the testing room for 30 min before the test. The nociceptive response or latency to respond to thermal stimuli is defined as the time (in seconds) taken from the moment the mouse is placed into the cylinder until it first licks its hind paws or is startled or jumps off the hot plate surface. To avoid thermal injury, the maximum time a mouse was allowed to remain on the hot plate was set at 20 s. Each mouse was tested once in each session to avoid thermal burn and to avoid substantial latency alterations from repeated assessments.

## Characterization of syngeneic mouse model of EM

### ELISA

The serum samples collected on day 12 following EM induction were used to measure Estrogen and cytokine levels to evaluate inflammation. To validate the model, we used an estrogen-specific ELISA because EM is an estrogen-dependent condition. This investigation was critical to demonstrate the estrogenic character of the model, as increasing estrogen levels encourage lesion survival and progression. Briefly, the samples were given time to clot at room temperature for 30 min. Then, centrifuged at 3000 × g for 15 min at 4 °C to separate the serum, and then stored at −80 °C until further analysis. Snap frozen serum samples were defrosted, and 0.1mL was used for cytokine measurement (pg/mL). Blood from the control mice (1x PBS and EB treatment) was used to determine baseline cytokine levels. A commercially available estradiol ELISA kit (ELK Biotechnology CO., LTD (Wuhan, China) #ELK8407)) was used for estrogen. Respective mouse-specific ELISA was employed to quantify inflammatory markers IL-6 (ABclonal, #RK00008), TNF-α (ABclonal, #RK00027), and TGF-β (ABclonal, #RP01458), and estrogen (ELK Biotechnology, #ELK8407). The estrogen, IL-6, TNF-α, and TGF-β ELISA were sensitive down to 4.28, 7.2, 6.5, and 3.9 pg/mL, respectively. The absorbance was measured at 450 nm using a microplate reader (MultiSkan FC Microplate Photometer with SkanIt software). The lack of statistically significant differences in some cases may have been pertaining to the small sample sizes. All procedures were conducted according to the kit guidelines.

### Hematoxylin and Eosin (H&E) staining

We utilized H&E staining to determine lesion structure as EM is diagnosed by the presence of viable endometrial-like glands and stroma outside of the uterus [28]. Briefly, sections (5 μm) placed on slides covered in Poly-L-lysine (PathnSitu Biotechnologies #PS011) were deparaffinized in xylene, rehydrated in descending grades of ethanol, stained with eosin Y (Sigma-Aldrich, #1.15935), followed by haematoxylin (Sigma-Aldrich, #HX03021349), and mounted using DPX mountant. A bright-field microscope Nikon Eclipse Ei 4 W, Nikon, Tokyo, Japan, was used to view the slides, and photos of representative areas were taken. The samples that did not reflect the morphology of the endometrium were not considered for any further investigation.

### IHC

To visually assess whether ectopic lesions comprised of proliferating epithelial cells, blood vessels, and macrophage immune population, ectopic lesions were immuno-stained with Ki67, CD31, and F4/80, respectively. In summary, ectopic lesions were serially paraffin sectioned, dewaxed in xylene, and rehydrated in descending order of ethanol gradients to distilled water. Antigens were recovered using a Tris-EDTA buffer at pH. 9, before antibody staining. The tissue sections were covered with peroxidase quencher, incubated for 10 min to block any endogenous peroxidase activity. The primary antibodies were added and incubated for 2 h at 4 °C. The expression was visualised using PolyExcel HRP/DAB Detection System-Two Step (PathnSitu Biotechnologies Pvt. Ltd, #PEH002) as per the manufacturer’s instructions. Slides were washed and counterstained with Methyl green (TCI chemicals, #M0498). Lastly, the slides were dehydrated, cleaned, dried, and mounted using DPX mountant (Sisco Research Laboratories Pvt Ltd. # 88147). Images were captured using a Nikon microscope (Nikon Eclipse Ei 4 W, Nikon, Tokyo, Japan) and analysed using ImageJ software (https://imagej.nih.gov/ij/download.html) [29]. The details of the primary and secondary antibodies, together with their manufacturer names and the concentrations employed in this study, are presented in Table 2.

**Table 2:** List of primary and secondary antibodies used for lesion characterization (DAB-IHC)

Table 2 Target	Primary Antibody	Species raised in	Dilution Used	Manufacturer & Catalog Number	RRID
Proliferating Cells (Ki67)	Anti-Ki67 (Monoclonal)	Rat	1:100	ThermoFisher Scientific, #14–5698−82	AB_10854564
Blood Vessels (CD31/PECAM)	Anti-CD31 (Monoclonal)	Rabbit	1:100	ThermoFisher Scientific, #14–0311−81	AB_467201
Macrophages (F4/80)	Anti-F4/80 (Monoclonal)	Rat	1:50	ThermoFisher Scientific, #14–4801−82	AB_467558
Rat IgG (H + L)	Goat anti-rat IgG (H + L) (HRP- conjugated)	Goat	1:1000	ThermoFisher Scientific, #A18865	AB_2535642

### FC

The PF was collected from the control and the EM mice in individual tubes. The mixture was treated with RBC lysis buffer (eBioscience™ 1X RBC Lysis Buffer #00–4333−57) for 15 min and centrifuged to eliminate erythrocytes. The cells were subsequently resuspended in DPBS for further analysis. A total of 1 × 10^6^ cells were seeded into each tube after cell counting and incubated with anti-MO-CD11b-Alexa flour 488 (eBioscience, #53–0112−80), anti-MO-CD 86-APC (eBioscience, #17–0862−81), and anti-MO-CD206-PE (eBioscience, #12–2061−80) antibodies at a concentration of 0.5 µg/test for 30 min at RT in the dark. The cells were then centrifuged to wash any unbound antibody, and the pellet was resuspended in 100 µl of DPBS for FC via a BD Accuri™ C6 Plus flow cytometer. The live and single cells were gated in unstained samples and were employed as a gating strategy for further samples. The color compensation for the samples was carried out via FlowJo software to obtain the percentage of positive cells in each channel as compared to the unstained. The details of the fluorescent conjugated primary antibodies, together with their manufacturer names and the concentrations, are presented in Table 3.

**Table 3:** List of antibodies used for FC

Table 3 Target	Primary Antibody	Species raised in	Dilution used	Manufacturer & Catalog Number	RRID
CD 11b Mouse	anti-Mo-CD 11b- Alexa flour 488 Monoclonal	Rat	0.5 µg/test	eBioscience #53–0112−80	AB_469901
CD 86 Mouse	CD86 (B7-2) Monoclonal Antibody (GL1), APC-eFluor™ 780	Rat	0.06 µg/test	eBioscience #17–0862−81	AB_469418
CD 206 Mouse	anti-Mo-Cd206 (MMR) Monoclonal Antibody (MR6F3), PE	Rat	0.125 µg/test	eBioscience #12–2061−80	AB_2637422
CD 68 Mouse	Anti-Mo-CD68 (FA-11), PE	Rat	0.25ug/test	eBioscience #12–0681−80	AB_2572569

## Statistical analysis

Statistical analysis and visualization were performed in GraphPad Prism 8.0.1, Inc. (GraphPad Inc., USA). Student’s t-tests or one-way ANOVA (Analysis of Variance) were used to compare the means associated with the study parameters across the groups. For ROC curve analysis and linear regression (LR), SPSS 30.0.0.0 was used. All graphical data are plotted as the means with standard errors of the means (SEMs). Statistical significance for all tests was established at *P* < 0.05 for data meeting the necessary assumptions for parametric analyses. Significance levels were as follows: * *P* < 0.05, ** *P* < 0.01, *** *P* < 0.001, **** *P* < 0.0001.

## Results

### Successful EM induction

**Fig. 2:**
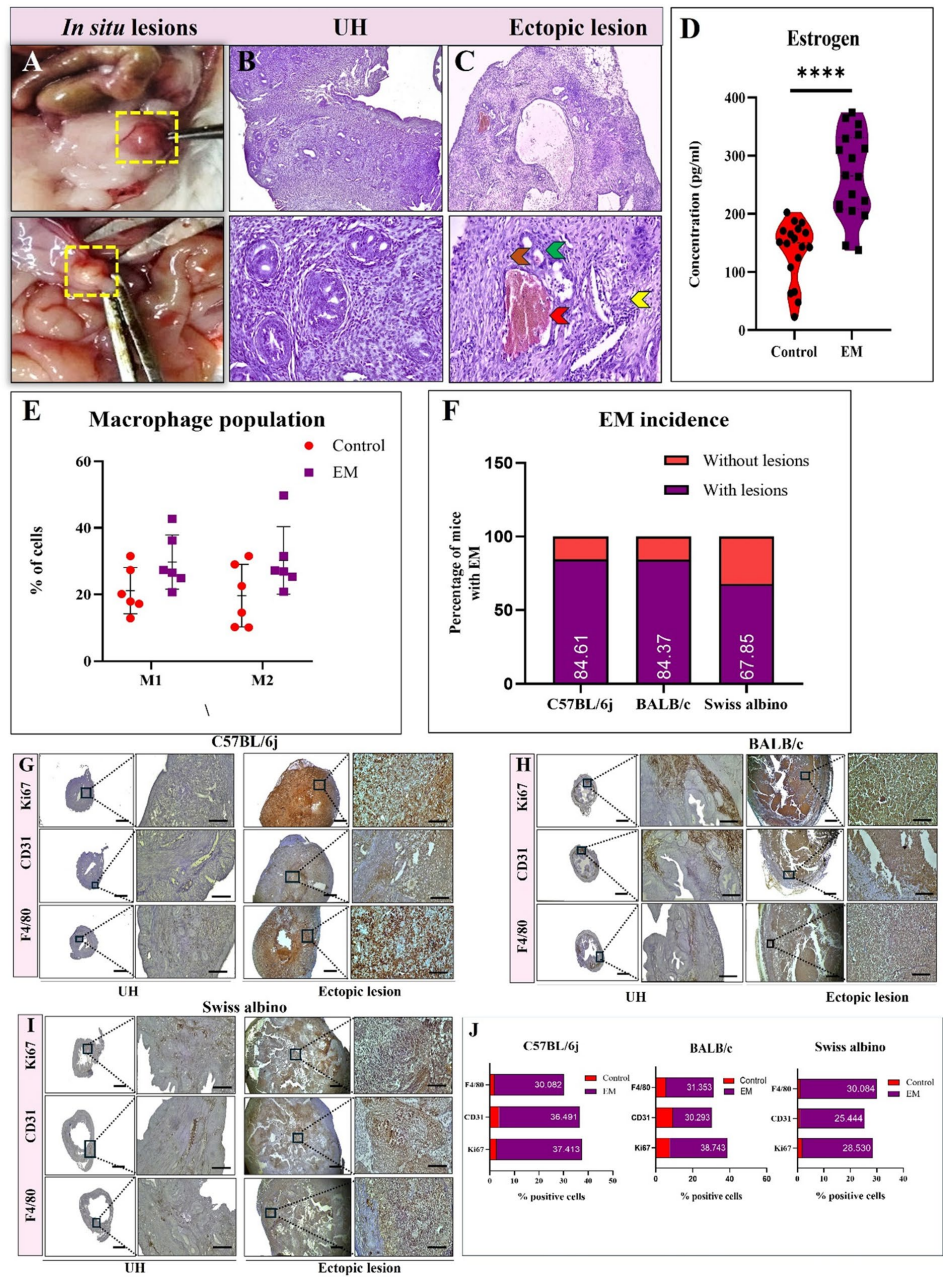
Lesion characterization and marker expression in an EM mice model. **A** Representative in situ gross images of ectopic lesions. The adhesions were white or red lesions with a varying degree of inflammation. **B** Representative H&E-stained images of UH from healthy control mice at estrous stage and the (**C**) obtained ectopic lesions from EM mice at day 12 post-UF injection. Ectopic lesions exhibited comparable anatomical characteristics to UH in situ. These included singular or multi-layered typical endometriotic glands (green arrow), well-differentiated heterogeneous stroma (yellow arrow), blood vessels (red arrow), including immune cells infiltration (brown arrow) (Magnification = 100x, Scale bar = 100 μm, Magnification = 400x, Scale bar = 20 μm). **D** Compared with control mice, EM mice presented with significantly higher estrogen levels in the blood (t value = 6.139, ***p value < 0.0001). **E** Analysis of the PF revealed a significantly greater proportion of anti-inflammatory M2 macrophages (*p value < 0.05) in EM mice than in controls (*n* = 6 for each group). **F** Bar graph representing the percentage incidence of EM in C57BL/6j, BALB/c, and Swiss albino mice. EM induction generated a success percentage of 84.61% for C57BL/6j, 84.37% for BALB/c, and 67.85% for Swiss albino. **G** and **J **- C57BL/6j EM mice expressed 37.41% of cells expressing Ki67 positive, a robust blood supply was apparent with 36.49% of cells demonstrating CD31 positivity, and 30.08% of cells exhibiting positive staining for F4/80. Similarly, (**H** and **J**)- BALB/c EM mice showed 38.7% Ki67 positive, 30.29% were CD31 positive, and 31.35% were F4/80 positive cells. **I** and **J**. And the Swiss albino EM mice showed 28.53% Ki67 positive cells, 25.4% were CD31 positive, and 30.08% F/80 positive cells

EM induction was carried out according to the schematic depiction presented in Fig. 1. All the experimental mice were returned to their home cages upon the injection with either UF (EM) or 1x PBS (control) and monitored for general health and changes in body weight until day 12. No recipients died, and there was no fluctuation in body weight, nor were there signs of internal hemorrhage or other adverse effects observed. Instead, the body weight of the recipients increased compared to the control group, and the overall health remained uncompromised, confirming that the UF injections did not have any negative impact on the recipient’s general well-being (Supplementary fig. S1). The gross observation of lesions showed the adhesions were either red or white with varying degrees of inflammation (Fig. 2A). The successful induction of EM was validated through histological characterisation, which confirmed the presence of endometrial glands (green), well differentiated heterogenous stroma (yellow), blood vessels (red arrow), including infiltrated immune cells (brown) (Fig. 2C). The UH in estrous stage of control mice is used as a reference (Fig. 2B). Lastly, compared with control mice, EM mice presented with significantly higher estrogen levels in the blood (t value = 6.139, p value < 0.0001****) (Fig. 2D). Analysis of the PF revealed a greater proportion of anti-inflammatory M2 macrophages in EM mice than in controls (*n* = 6 for each group) (Fig. 2E and Supplementary fig. S2). The lesions from all 3 strains were further characterized by positive immunostaining for anti-Ki67 (proliferation marker), anti-CD31 (endothelial marker), and anti-F4/80 (macrophage marker): C57BL/6j (Fig. 2F and I), BALB/c (Fig. 2G and I), Swiss albino (Fig. 2H and I). Ectopic lesions of EM mice from all three strains exhibited elevated Ki67, CD31, and F4/80 expression compared to the UH tissue from control mice. The percent lesion incidence was calculated for all three strains, accounting for C57BL/6j (84.61%), BALB/c (84.37%), and Swiss albino (67.85%), respectively (Fig. 2F).

### EM-induced anxiety/chronic pain significantly decreased burrowing behavior in a syngeneic EM mouse model

**Fig. 3:**
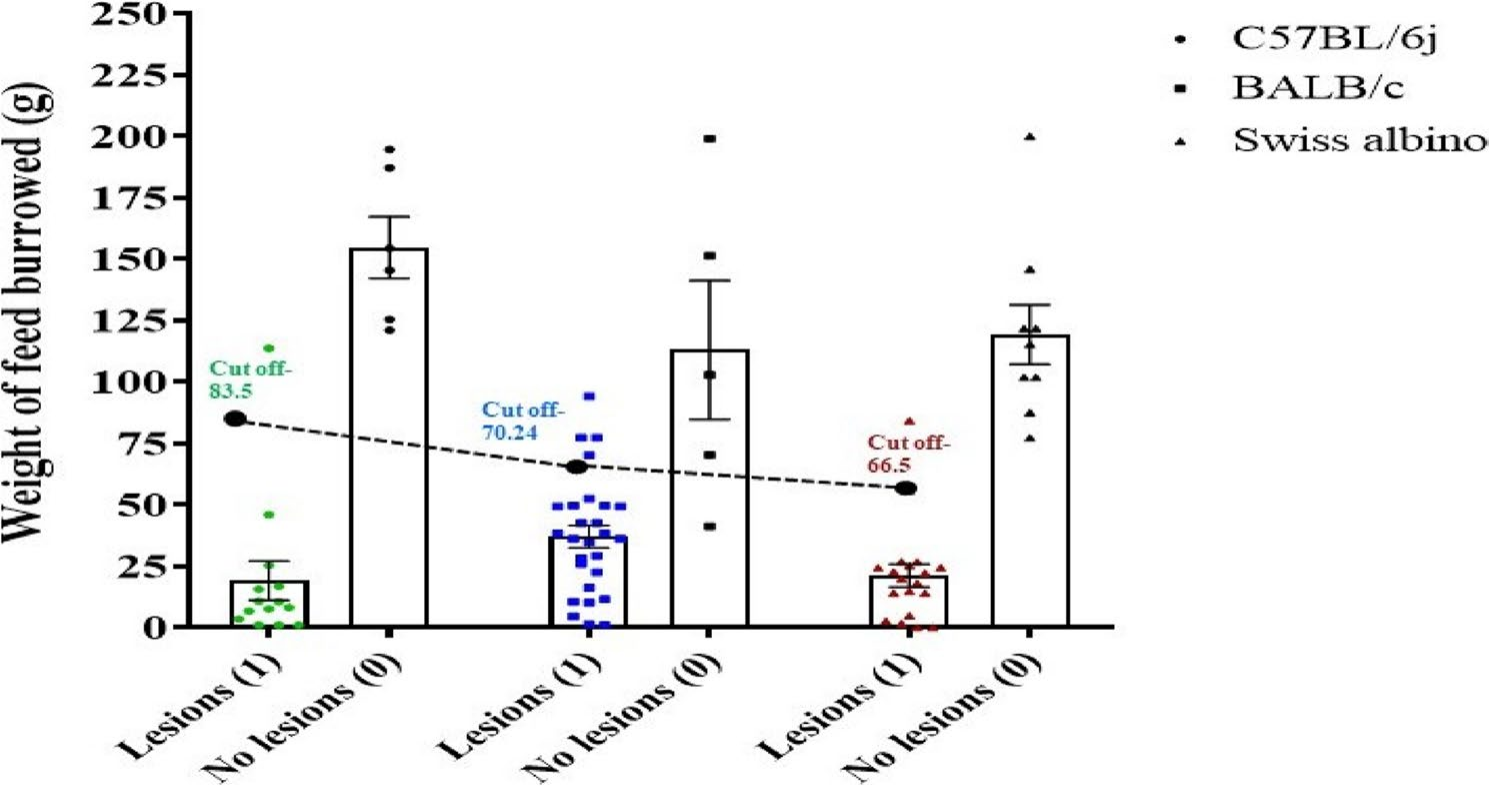
Comparison of overnight feed burrowed across 3 strains. The graph represents the comparison of burrowing overnight values used for the ROC analysis (EM mice with lesions vs. EM mice without lesions) for each strain, indicating the cut-off value, calculated by the Youden´s index. Each data point represents the amount of feed burrowed in grams, color-coded by strain: C57BL/6j (green), BALB/c (blue), and Swiss albino (brown). (C57BL/6j- with lesions, *n* = 14, without lesions, *n* = 6, BALB/c- with lesions, *n* = 27, without lesions, *n* = 5, Swiss albino- with lesions, *n* = 19, without lesions, *n* = 9)

In clinical research, the visual analog scale (VAS) is frequently employed to evaluate pain quantitatively. As rodents do not vocalize their pain, examination can be performed via behavioral testing. Thus, to evaluate behavioral changes as an indirect measure of pain or discomfort, we conducted a BA before euthanizing the EM recipient mice. ROC curve analysis was performed to evaluate the diagnostic reliability of the burrowing pattern for EM incidence. Table 4 represents information about strain-specific details obtained, including the lesion incidence (1-with lesion, 0-without lesion), cut-off value (Overnight burrowed pellets in g), Sensitivity, 1-Specificity, AUC (Youden’s Index), and the overall model quality. The comparison of overnight burrowing values used for the analysis is shown in Fig. 3.

**Table 4:** Strain-specific diagnostic parameters for the burrowing assay (BA) in predicting EM incidence. The column “With lesions (Disease state 1)” refers to the lesion-positive EM recipient mice. The column “Without lesions (Disease state 0: no lesion + Control)” refers to the total number of animals without ectopic lesions used in the ROC analysis, which comprises both the lesion-negative HB recipient mice and the respective strain controls

Strain	With lesions (Disease state 1)	Without lesions (No lesion + Control) (Disease state 0)	Cut off value (Overnight burrowed pellets in g)	Sensitivity	1-Specificity	AUC (Youden’s Index)	Overall model quality
C57BL/6j	14	12 (6 HB + 6 Control)	83.5	1	0	1	1
BALB/c	27	30 (5 HB + 25 Control)	70.245	0.889	0.2	0.689	0.74
Swiss albino	19	29 (9 HB + 20 Control)	66.5	0.947	0	0.947	0.98
All strains	60	80 (20 HB + 56 Control)	70.245	0.933	0.05	0.883	0.95

**Fig. 4:**
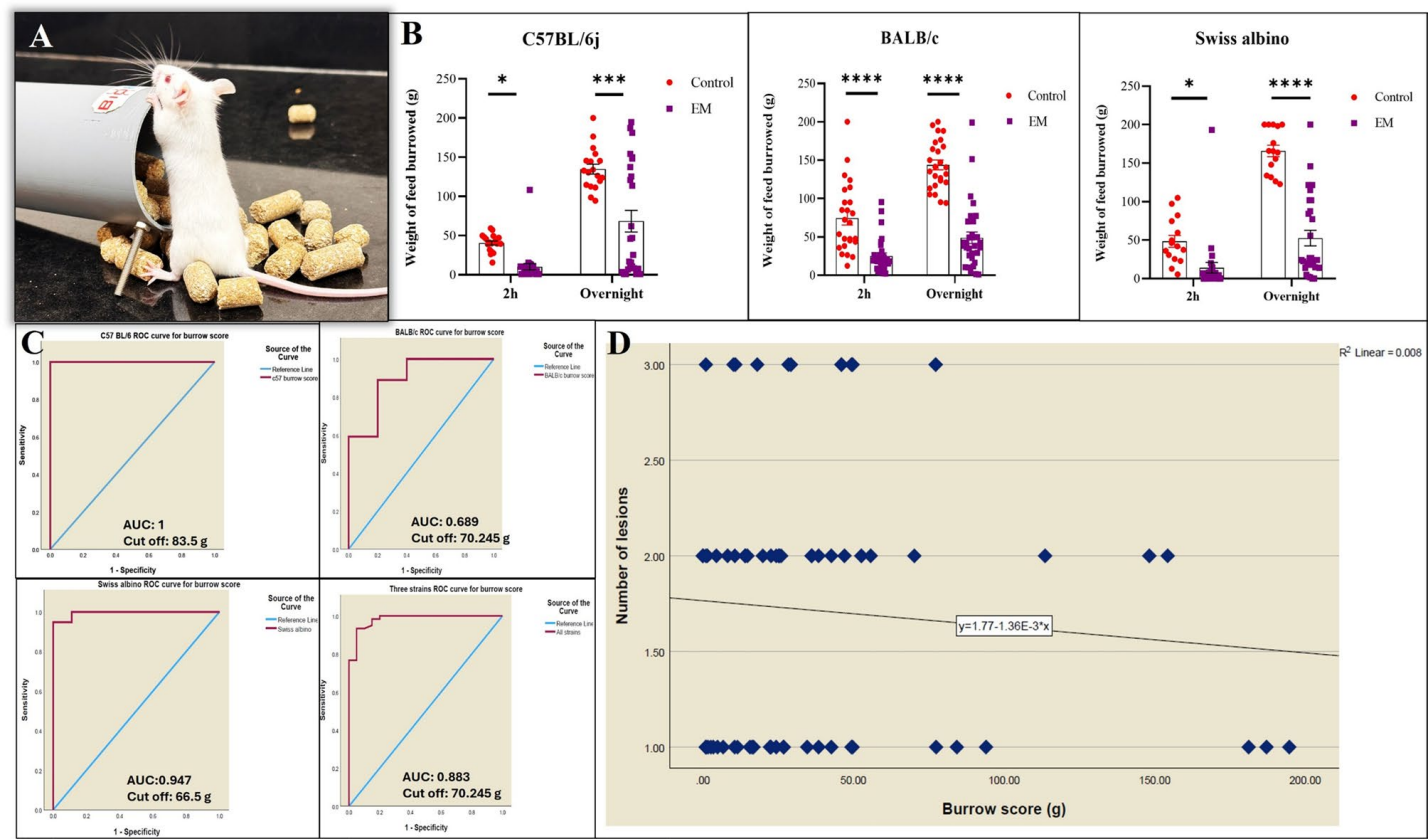
Burrowing behavior of 3 mouse strains post-induction. **A** Representative image of a standard BA setup. **B** Quantification of burrowing activity in EM and control mice across three different strains: C57BL/6j (EM *n* = 26, Control *n* = 11), BALB/c (EM *n* = 32, Control *n* = 25), and Swiss albino (EM *n* = 28, Control *n* = 20). Bar graphs depict the mean ± SEM of substrate (grams) removed after 2 h and overnight. Statistical significance was determined by two-way ANOVA with Sidak’s multiple comparison test (*p value < 0.05, ***p value < 0.001, ****p value < 0.0001). **C** ROC curve analysis of burrowing scores for predicting the presence of lesions in each strain: C57BL/6j (AUC = 1, cutoff burrow score = 83.5 g, sensitivity = 1, 1-specificity = 0, overall model quality = 1), BALB/c (AUC = 0.689, cutoff burrow score = 70.24 g, sensitivity = 0.889, 1-specificity = 0.2, overall model quality = 0.74), and Swiss albino (AUC = 0.947, cutoff burrow score = 66.5 g, sensitivity = 0.947, 1-specificity = 0, overall model quality = 0.98). Combined ROC curve analysis of burrowing scores for all three strains (AUC = 0.883, cutoff burrow score = 70.24 g, sensitivity = 0.933, 1-specificity = 0.05, overall model quality = 0.95). **D** LR analysis illustrating a negative correlation between burrowing score and the number of EM lesions across all three strains. The solid line represents the LR fit

The burrowing setup is shown in Fig. 4A. The EM mice demonstrated a significant decrease in burrowing behavior compared to the control across all three strains. The C57BL/6j EM mice (*n* = 26), BALB/c EM mice (*n* = 32), and Swiss albino EM mice (*n* = 28) showed a significant decrease in burrowing activity at 10.387 g ± 4.19 g (*p value < 0.05, t value = 2.651), 25.1 g ± 3.87 g (****p value < 0.0001, t value = 5.114) and 14.3 g ± 6.8 g (*p value < 0.05, t value = 2.5) compared to respective strains control mice at 58.22 g ± 15.9 g, 74.48 g ± 9 g and 43.28 g ± 6.6 g after 2 h. A similar trend was observed at overnight time points at 68.337 g ± 13.8 g (***p value < 0.0001, t value = 4.172) for C57BL/6j, 48.9 g ± 7.4 g (****p value < 0.0001, t value = 9.825) for BALB/c and 52.68 g ± 10 g (****p value < 0.0001, t value = 9.677) as compared to respective control at 145.77 g ± 8.4 g, 143.76 g ± 6.3 g and 164.8 g ± 6.35 g indicating the presence of anxiety or pain (Fig. 4B). Based on burrowing scores in EM mice, we identified two distinct groups: EM mice with low burrow score (LB), and those with high burrow score (HB). Notably, burrowing behavior was not significantly correlated with the number of lesions across all three strains (r² = 0.008) with a negative slope.

### Burrowing behavior is predictive of EM incidence

For C57BL/6j mice, the ROC curve revealed an area under the curve (AUC) of 1, reflecting 100% sensitivity and a 1-specificity of 0, indicating an excellent model with a cut-off value of 83.5 g and an overall model quality of 1.00. Similarly, the ROC curve for BALB/c mice produced an AUC of 0.689, with a sensitivity of 0.889, a 1-specificity of 0.2, and a cut-off value of 70.245 g, leading to an overall model quality of 0.74. The Swiss albino strain had an AUC of 0.947, a sensitivity of 0.947, a 1-specificity of 0, and a cut-off value of 66.5 g, resulting in an overall model quality of 0.98. When data from all three strains were combined, the overall ROC curve analysis yielded an AUC of 0.883, a sensitivity of 0.933, a 1-specificity of 0.05, and a cut-off value of 70.245 g, resulting in an overall model quality of 0.95 **(**Fig. 4C**).** The ROC revealed a Youden’s index of 1, 0.689, and 0.947 for C57BL/6j, BALB/c, and Swiss albino, respectively, indicating the burrow score is an effective non-invasive test for EM prediction. Based on the ROC curve, recipients burrowing less than the established cut-off weight were classified as low burrowers and, hence, EM. Notably, burrowing behavior was not significantly correlated with the number of lesions across all three strains (r² = 0.008) with a negative slope (Fig. 4D). Table 4 represents information about strain-specific details obtained including the lesion incidence (1-with lesion, 0-without lesion), cut off value (Overnight burrowed pellets in g), Sensitivity, 1-Specificity, AUC (Youden’s Index), and the overall model quality.

### The LB-EM mice exhibited altered behavioral responses

It has been suggested that combining evoked responses to assess the global impact of lesion presence with non-evoked behavioral patterns, such as burrowing, may enhance translational relevance [30]. Therefore, we investigated whether there were any other behavioral changes in the LB-EM mice and observed substantial changes.

#### LB-EM mice showed diminished exploratory behavior

**Fig. 5:**
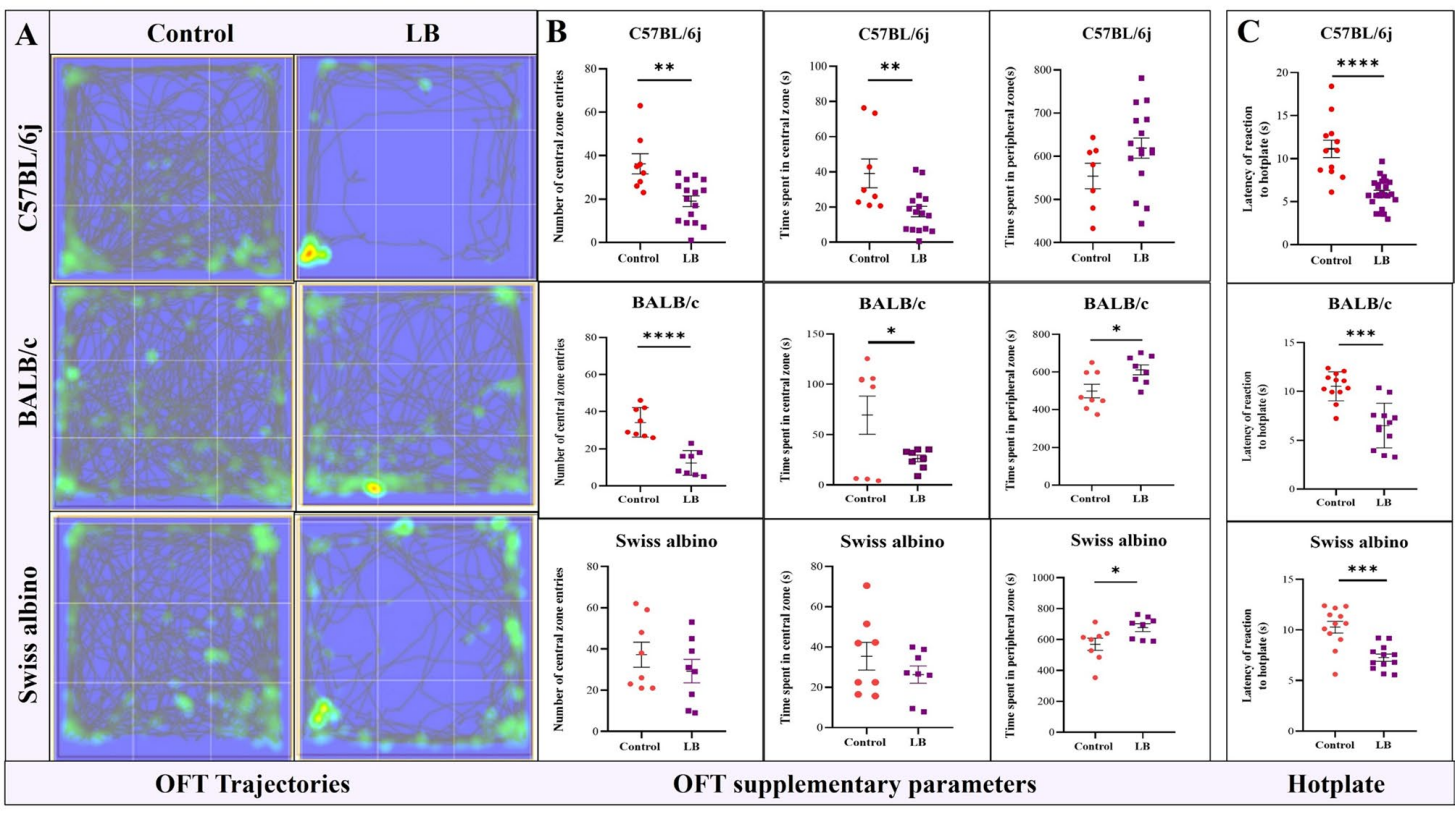
OFT trajectory and Hotplate behavior across 3 strains. EM impairs exploratory behavior and enhances pain sensitivity in LB mice. **A** Representative track plots and superimposed heat maps illustrating the diminished exploratory behavior across all 3 strains of LB mice (**B**) Bar graphs representing the additional OFT parameters: Reduced number of central entries: LB mice exhibited a significantly lower number of central entries compared to controls in C57BL/6j [19 ± 2.43 vs. 36.25 ± 4.628 (**p < 0.01, t = 3.65)], BALB/c [12.38 ± 2.367 vs. 34.25 ± 2.776 (****p < 0.0001, t = 5.996)], and Swiss albino [29.25 ± 5.697 vs. 37.25 ± 6.064 (p = not significant, t = 0.9615)]. Reduced time spent in central zone (s): The time spent in the central zone was significantly reduced in LB mice compared to controls in C57BL/6j [17.51 ± 2.94 vs. 39.14 ± 8.203 (**p = 0.01, t = 3.065)], BALB/c [26.41 ± 3.372 vs. 69.28 ± 18.88 (*p = 0.05, t = 2.234)], and Swiss albino [26.32 ± 4.316 vs. 35.43 ± 6.892 (p = not significant, t = 1.12)]. Increased time spent in peripheral zone (s): Conversely, the time spent in the peripheral zone was significantly greater in LB mice compared to controls in C57BL/6j [618.69 ± 23.29 vs. 554.3 ± 29.42], BALB/c [611.1 ± 26.39 vs. 498.4 ± 361.6 (*p < 0.05, t = 2.517)], and Swiss albino [677.39 ± 25.15 vs. 569.7 ± 39.33 (*p < 0.05, t = 2.308)]. (C57BL/6j: Control *n* = 8, LB *n* = 16; BALB/c and Swiss albino: Control *n* = 8, LB of thermal hyperalgesia using the hot plate test: Bar graphs show the significantly reduced latency to respond to thermal stimuli in LB mice compared to controls for C57BL/6j (5.985 ± 0.3397 s vs. 11.14 ± 1.007 s, ****p value < 0.0001, t value = 6.045), BALB/c (6.515 ± 0.6565 s vs. 10.53 ± 0.4281 s, ****p value < 0.0001, t value = 5.124), and Swiss albino (7.265 ± 0.3528 s vs. 10.28 ± 0.5806 s, ***p value < 0.001, t value = 4.434) mice. Data represented as mean ± SEM, and *p* values were generated from C57BL/6j: Control *n* = 12, LB *n* = 24; BALB/c and Swiss albino: Control *n* = 12, LB *n* = 12 per group. Statistical significance was determined by unpaired t-test (*p value < 0.05, **p value < 0.01, ***p value < 0.001, ****p value < 0.0001

The OFT divides the area into central and peripheral zones. Mice that experience greater pain or anxiety tend to spend more time in the peripheral zone while avoiding the central zone. The trace trajectories represent the overall distance explored by each mouse, which indicates motor activity. The OFT results in Fig. 5A and Supplementary Table 1 revealed that LB mice showed diminished exploratory behavior as represented by the track plots. Figure 5B shows the significantly reduced number of central entries in LB mice compared to controls for C57BL/6j [19 ± 2.43 vs. 36.25 ± 4.628 (**p value < 0.01, t value = 3.65)], BALB/c [12.38 ± 2.367 vs. 34.25 ± 2.776 (****p value < 0.0001, t value = 5.996)], and Swiss albino [29.25 ± 5.697 vs. 37.25 ± 6.064 (*p* value not significant, t value = 0.9615)]. Similarly, the time spent in the central zone was significantly reduced in LB mice [C57BL/6j: 17.5125 s ± 2.94 s vs. 39.14 s ± 8.203 s, (**p value = 0.01, t value = 3.065); BALB/c: 26.41 s ± 3.372 s vs. 69.28 s ± 18.88 s (*p value = 0.05, t value = 2.234); Swiss albino: 26.32 s ± 4.316 s vs. 35.43 s ± 6.892 s (p value = not significant, t value = 1.12)] compared to the control. Conversely, the time spent in the peripheral zone was significantly greater in LB mice [C57BL/6j: 618.693 s ± 23.29 s vs. 554.3 ± 29.42 s; BALB/c: 611.1 s ± 26.39 s vs. 498.4 s ± 361.6 s (*p value < 0.05, t value = 2.517; Swiss albino: 677.39 s ± 25.15 s vs. 569.7 s ± 39.33 (*p value < 0.05], t value = 2.308] compared to the control. Data represented as mean ± SEM and the t value and p values generated by the unpaired t test from C57BL/6j control, *n* = 8, and LB, *n* = 16, BALB/c and Swiss albino control, *n* = 8, and LB, *n* = 8. *p value < 0.05, **p value < 0.01, ****p value < 0.0001 represent statistically significant values.

#### LB-EM mice showed increased thermal sensitivity to heat

Since both mice with induced EM and women with EM exhibit generalized nociception and because mice do not vocalize distress [31], hotplate latency can be used as a surrogate measure of the severity of generalized hyperalgesia related to EM. As shown in Fig. 5C, the hot plate test revealed increased thermal sensitivity in EM mice across all three strains The latency to respond to thermal stimuli was significantly shorter in LB mice compared to controls (C57BL/6j: 5.985 ± 0.3397 s vs. 11.14 ± 1.007 s, ****p value < 0.0001, t value = 6.045; BALB/c: 6.515 ± 0.6565 s vs. 10.53 ± 0.4281 s, ****p value < 0.0001, t value = 5.124; Swiss albino: 7.265 ± 0.3528 s vs. 10.28 ± 0.5806 s, ***p value < 0.001, t value = 4.434). Data reflect mean ± SEM and t value and p value generated by the unpaired t test from (C57BL/6j, control, *n* = 12, and LB, *n* = 24, BALB/c and Swiss albino, control and LB, *n* = 12 each). The t-value and p-value generated by the unpaired t test. *p value < 0.05, **p value < 0.01, ***p value < 0.001 ****p value < 0.0001.

### HB-EM mice did not exhibit altered behavioral responses

We also investigated whether there were any other behavioral changes in the HB-EM mice.

#### The HB-EM mice exhibited normal exploratory behavior

**Fig. 6:**
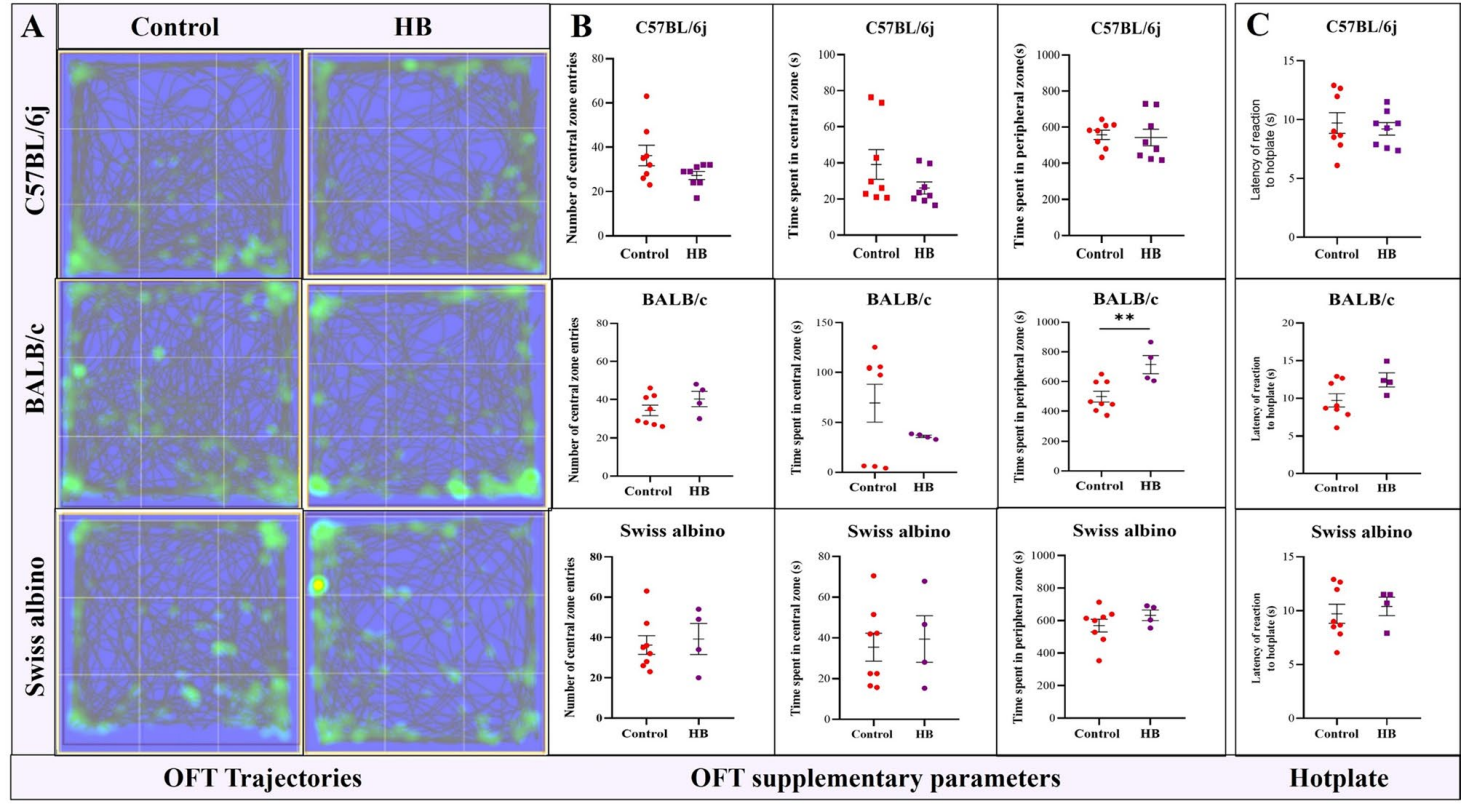
EM does not alter exploratory behavior and pain sensitivity in HB mice. **A** Representative track plots and superimposed heat maps illustrating the exploratory behavior across all 3 strains of HB-EM mice (**B**) Bar graphs representing the additional OFT parameters: Number of central entries: HB-EM mice exhibited a comparable number of central entries in C57BL/6j [27.25 ± 4.984 vs. 36.25 ± 4.628 (p = not significant, t = 1.806)], a slight increase in BALB/c [40.25 ± 4.838 vs. 34.25 ± 2.776 (p = not significant, t = 1.24)], and comparable in Swiss albino [39.25 ± 10.19 vs. 36.25 ± 6.064 (p = not significant, t = 0.196] compared to controls. Time spent in central zone (s): The time spent in the central zone in comparable in C57BL/6j [26.13 ± 8.847 vs. 39.14 ± 8.203 (p = not significant, t = 1.471)], a slight decrease in BALB/c [39.25 ± 27.85 vs. 69.28 ± 18.88 (p = not significant, t = 1.078)], and comparable in Swiss albino [39.45 ± 12.59 vs. 35.43 ± 6.892 (p = not significant, t = 0.319)] compared to controls. Time spent in peripheral zone (s): Conversely, the time spent in the peripheral zone was comparable in C57BL/6j [557.9 ± 64.19 vs. 543.07 ± 120.81 (p = not significant, t = 0.7152)], a significant increase in BALB/c [688.5 ± 59.73 vs. 498.4 ± 361.6 (**p < 0.01, t = 3.18)], and comparable in Swiss albino [632.8 ± 61.04 vs. 569.7 ± 39.33 (p = not significant, t = 1.034)] compared to controls. (C57BL/6j control *N*=8, HB *N*=*8*; BALB/c and Swiss albino: Control *n* = 8, HB *n* = 4 per group). Significance levels: *P < 0.05, **P < 0.01, ****P < 0.0001. **C** Assessment of thermal hyperalgesia using the hot plate test: Bar graphs show the comparable latency to respond to thermal stimuli in HB-EM mice compared to controls for C57BL/6j (9.209 ± 1.029 s vs. 11.14 ± 1.007 s, p value = not significant, t value = 0.4838), BALB/c (6.515 ± 0.6565 s vs. 10.53 ± 0.4281 s, ****p value < 0.0001, t value = 5.124), and Swiss albino (7.265 ± 0.3528 s vs. 10.28 ± 0.5806 s, ***p value < 0.001, t value = 4.434). Data represented as mean ± SEM, and p values were generated from C57BL/6j: Control *n* = 8, HB *n* = 8; BALB/c and Swiss albino: Control *n* = 8, HB *n* = 4 per group. Statistical significance was determined by unpaired t-test (*p value < 0.05, **p value < 0.01, ***p value < 0.001, ****p value < 0.0001)

The OFT results in Fig. 6A, Supplementary Table 2 demonstrated that HB mice had comparable exploratory behavior to that of controls, as represented by the track plots. Figure 6 shows the central entries were similar between the HB and control mice for C57BL/6j [27.25 ± 4.984 vs. 36.25 ± 4.628 (p = not significant, t = 1.806)], and Swiss albino [39.25 ± 10.19 vs. 37.25 ± 6.064, (p = not significant, t = 0.196)], BALB/c exhibited a slight but non-significant increase [40.25 ± 4.838 vs. 34.25 ± 2.776 (p = not significant, t = 1.24)], and comparable in Swiss albino [39.25 ± 10.19 vs. 36.25 ± 6.064 (p = not significant, t = 0.196] compared to controls. The duration spent in the central zone was similar for C57BL/6j mice [26.13 ± 8.847 vs. 39.14 ± 8.203 (p = not significant, t = 1.471)], and Swiss albino mice [39.45 s ± 12.59 s vs. 35.43 s ± 6.892 s, (p = not significant, t = 0.319)], whereas BALB/c mice exhibited a slight, non-significant decrease [39.25 s ± 27.85 s vs. 69.28 s ± 18.88 s, (p = not significant, t = 1.078)]. In contrast, the duration in the peripheral zone was comparable for C57BL/6j [557.9 ± 64.19 vs. 543.07 ± 120.81 (p = not significant, t = 0.7152)], a significant increase in BALB/c [688.5 ± 59.73 vs. 498.4 ± 361.6 (**p < 0.01, t = 3.18)], and comparable in Swiss albino [632.8 ± 61.04 vs. 569.7 ± 39.33 (p = not significant, t = 1.034)] compared to controls. Collectively, these results suggest that HB-EM mice maintained exploratory patterns similar to their respective controls, with no consistent decrease or increase across groups and only minor, non-significant strain-specific variations, especially in BALB/c mice. Data reflect mean ± SEM and t value and p value generated by the unpaired t-test from C57BL/6j: control *n* = 8, HB *n* = 8; BALB/c and Swiss albino: control *n* = 8, HB *n* = 4 by unpaired t-test. *p value < 0.05, **p value < 0.01, ***p value < 0.001 ****p value < 0.0001.

#### HB-EM mice showed normal thermal sensitivity to heat

Assessment of thermal hyperalgesia in HB mice using the hot plate test: As shown in Fig. 6C, the hot plate test exhibited a comparable latency to respond to thermal stimuli in HB-EM mice. The latency to respond was comparable in all groups of HB-EM mice compared to controls (C57BL/6j: 9.209 ± 1.029 s vs. 11.14 ± 1.007 s, *p* value = not significant, t value = 0.4838), but significantly shorter in BALB/c (BALB/c: 6.515 ± 0.6565 s vs. 10.53 ± 0.4281 s, p value = not significant, t value = 1.925) and comparable in Swiss albino (7.265 ± 0.3528 s vs. 10.28 ± 0.5806 s, ***p value < 0.001, t value = 4.434). Data are reported as mean ± SEM, and p values were generated from C57BL/6j: control *n* = 8, HB *n* = 8; BALB/c and Swiss albino: control *n* = 8, HB *n* = 4 per group. Statistical significance was assessed by unpaired t-test (*p value < 0.05, **p value < 0.01, ***p value < 0.001, ****p value < 0.0001).

### Correlation between burrowing behavior and other behavioral assays in LB mice

**Fig. 7:**
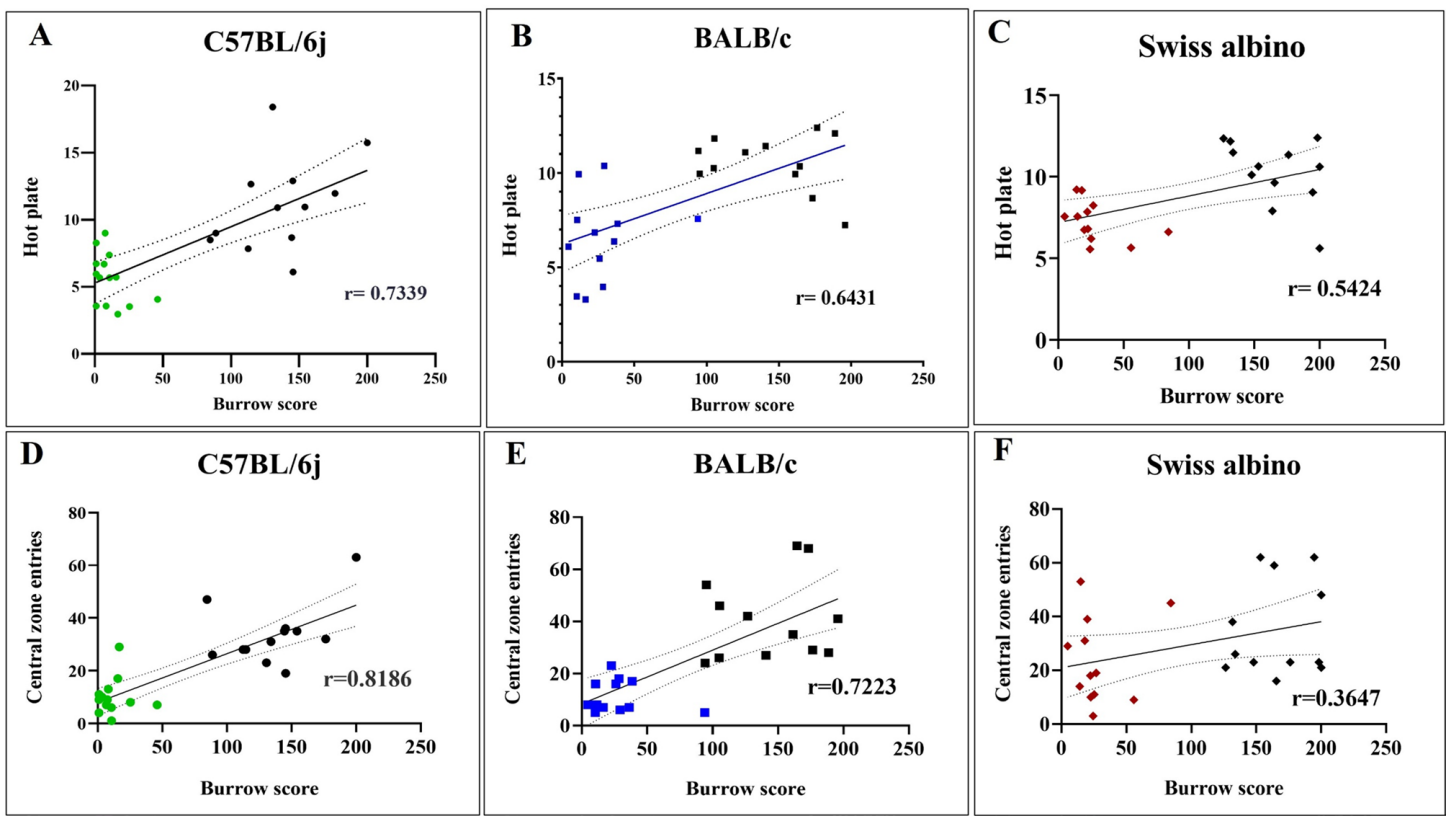
Correlation between burrow score and hotplate sensitivity. Correlation plots illustrate the relationship between BA performance (grams of feed burrowed) and thermal latency, and BA performance (grams of feed burrowed) and central zone entries across three mouse strains. Each data point represents an individual mouse, color-coded by strain: C57BL/6j (green), BALB/c (blue), and Swiss albino (brown). **A** Positive correlation between burrowing behavior and pain sensitivity in LB EM mice: Pearson’s correlation analysis revealed a significantly high correlation between burrowing activity and thermal sensitivity in C57BL/6j (*r* = 0.7339, **** *p* value < 0.0001) (**B**) BALB/c mice showed a high correlation with thermal sensitivity (*r* = 0.6431, ***p value < 0.001) (**C**) Further, the Swiss albino exhibited a moderate positive correlation with thermal sensitivity (*r* = 0.5424, **p value < 0.01). **D** Pearson’s correlation analysis revealed a very highly significant correlation between burrowing activity and central zone entries in C57BL/6j (*r* = 0.8186, **** *p* value < 0.0001). **E** BALB/c mice showed a high positive correlation with central zone entries (*r* = 0.7223, ****p value < 0.0001). **F** Further, the Swiss albino exhibited a low correlation with central zone entries (*r* = 0.3647, p value = not significant). The correlation plot was generated using Control *n* = 12 and LB *n* = 14 for C57BL/6j, and Control *n* = 12 and LB *n* = 12 for BALB/c and Swiss albino

We used Pearson’s correlation analysis to evaluate the linear relationship between thermal and exploratory activity with burrowing activity in LB-EM mice. Pearson’s correlation analysis revealed a significant strong positive correlation between burrowing activity and thermal sensitivity (hot plate latency) and exploratory behavior (OFT) in C57BL/6j (*r* = 0.734, ***p value < 0.001), (*r* = 0.844, ***p value < 0.001) and (*r* = 0.909, ***p value < 0.001) respectively (Fig. 7A). While BALB/c mice showed a moderate positive correlation with thermal sensitivity (*r* = 0.643, ***p value < 0.001), a strong correlation with exploratory behavior (*r* = 0.862, ***p < 0.001) (Fig. 7B). Further, Swiss albino exhibited a weak positive correlation with thermal sensitivity (*r* = 0.542, **p value < 0.01); but a strong positive correlation with exploratory behavior (*r* = 0.896, ***p value < 0.001) (Fig. 7C) (Control *n* = 12 and LB *n* = 12 for all three strains). Pearson’s correlation analysis revealed a significantly high correlation between burrowing activity and central zone entries in C57BL/6j (*r* = 0.8186, **** *p* value < 0.0001). (E) BALB/c mice showed a high positive correlation with central zone entries (*r* = 0.7223, ****p value < 0.0001). (F). Further, the Swiss albino exhibited a low correlation with central zone entries (*r* = 0.3647, ^ns^*p* value = 0.07). (C57BL/6j: Control *n* = 12 and LB *n* = 14, BALB/c: Control *n* = 12 and LB *n* = 12, Swiss albino: Control *n* = 12 and LB *n* = 12). This shows that when the burrowing activity is reduced, there is a corresponding reduction in sensitivity to thermal stimuli and exploratory behavior.

### Correlation between burrowing behavior and other behavioral assays in HB mice

**Fig. 8:**
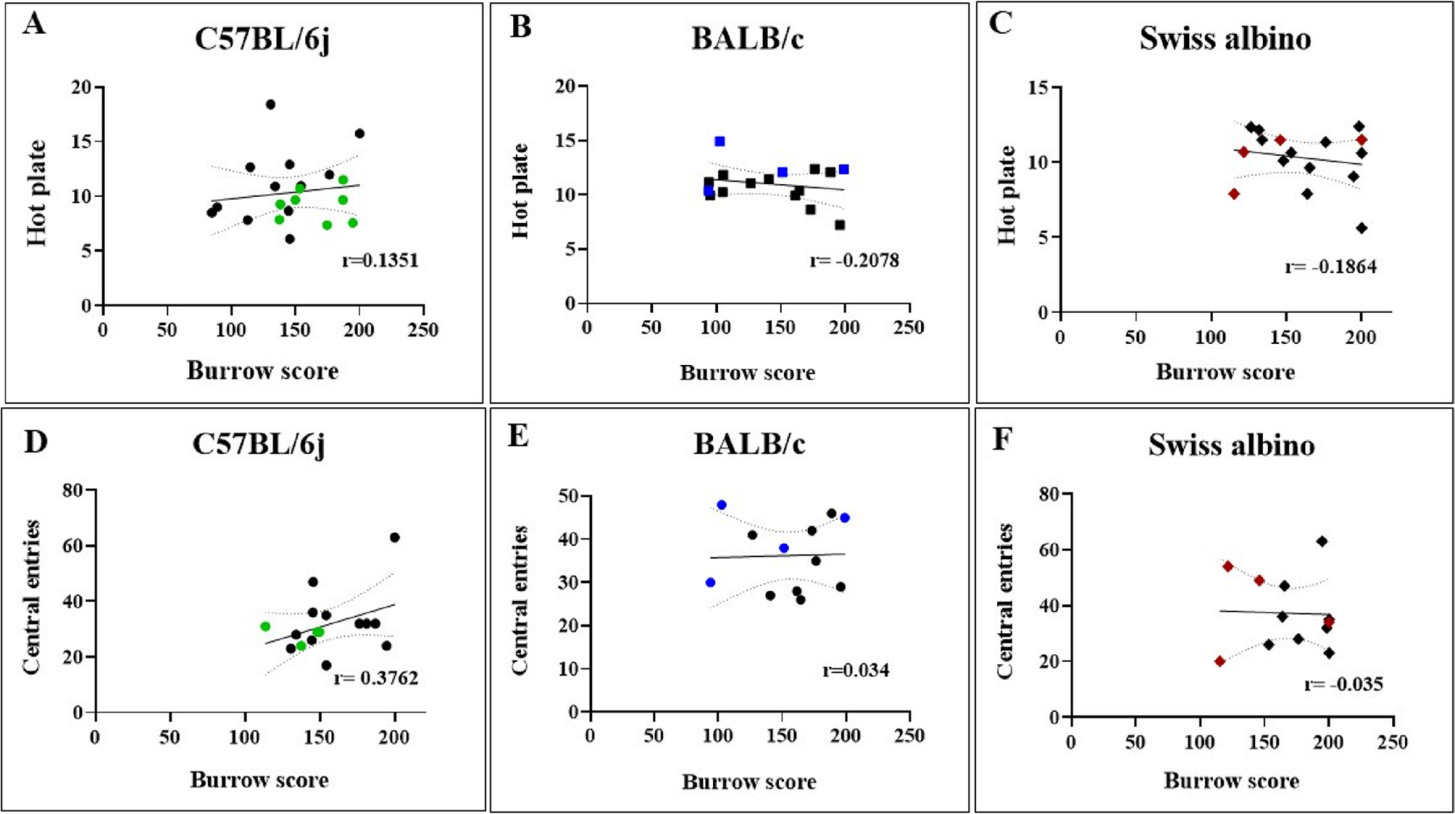
The correlation between burrowing behavior and pain sensitivity in HB mice. Correlation plots illustrate the relationship between BA performance (grams of feed burrowed) and thermal latency, and BA performance (grams of feed burrowed) and central zone entries across three mouse strains. Each data point represents an individual mouse, color-coded by strain: C57BL/6j (green), BALB/c (blue), and Swiss albino (brown). **A** Pearson’s correlation analysis revealed a very weak correlation between burrowing activity and thermal sensitivity in C57BL/6j (*r* = 0.1351, *p* value = not significant). **B** BALB/c mice showed a weak negative correlation with thermal sensitivity (*r* = −0. 2078, p value = not significant). **C** Further, the Swiss albino exhibited a very weak negative correlation with thermal sensitivity (*r* = −0.1864, **p value < 0.01). The correlation plot was generated using Control *n* = 12 and HB *n* = 8 for C57BL/6j, and Control *n* = 12 and HB *n* = 4 for BALB/c and Swiss albino. **D** Pearson’s correlation analysis revealed a weak positive correlation between burrowing activity and central zone entries in C57BL/6j (*r* = 0.3762, **** *p* value < 0.0001). **E** BALB/c mice showed a very weak positive correlation with central zone entries (*r* = 0.034, ****p value < 0.0001). Control N=8, HB N=*4* for C57BL/6j. **F** Further, Swiss albino exhibited a very weak negative correlation with central zone entries (*r* = 0.3647, *p* value = not significant). The correlation plot was generated using Control *n* = 8 and HB *n* = 4 for C57BL/6j, and Control *n* = 8 and HB *n* = 4 for BALB/c and Swiss albino

We used Pearson’s correlation analysis to evaluate the linear relationship between thermal and exploratory activity with burrowing activity in HB-EM mice. Pearson’s correlation analysis revealed a very weak correlation between burrowing activity and thermal sensitivity in C57BL/6j (*r* = 0.1351, *p* value = not significant) (Fig. 8A). BALB/c mice showed a weak negative correlation with thermal sensitivity (*r* = − 0.2078, *p* value = not significant) (Fig. 8B). Further, Swiss albino exhibited a very weak negative correlation with thermal sensitivity (*r* = − 0.1864, **p value < 0.01) (Fig. 8C). Correlation between burrowing activity and exploratory behavior, as assessed by central zone entries, revealed a weak positive correlation in C57BL/6j (*r* = 0.3762, ****p value < 0.0001) (Fig. 8D). BALB/c mice showed a very weak positive correlation (*r* = 0.034, ****p value < 0.0001) (Fig. 8E), whereas Swiss albino exhibited a very weak negative correlation (*r* = − 0.3647, *p* value = not significant) (Fig. 8F).The correlation plots were generated using Control *n* = 8 and HB *n* = 8 for C57BL/6j, and Control *n* = 8 and HB *n* = 4 for BALB/c and Swiss albino.

## Group stratification based on the burrowing behavior

Post-dissection, to validate the impact of burrowing, animals were classified into low burrow score with lesions (LB+) and high burrow score without lesions (HB-) categories based on burrowing score and lesion presence. Among EM-induced mice, every LB mouse had at least one ectopic lesion. Importantly, the burrowing assay demonstrated a near-perfect classification accuracy in differentiating between lesion-positive and lesion-negative EM mice, since none of the EM-induced mice exhibiting burrowing performance above the diagnostic threshold were found to have lesions following dissection. This binary categorization further strengthens the translational potential of burrowing as an early incidence indicator.

## LB + mice showed enhanced pro-inflammatory cytokine expression

**Fig. 9:**
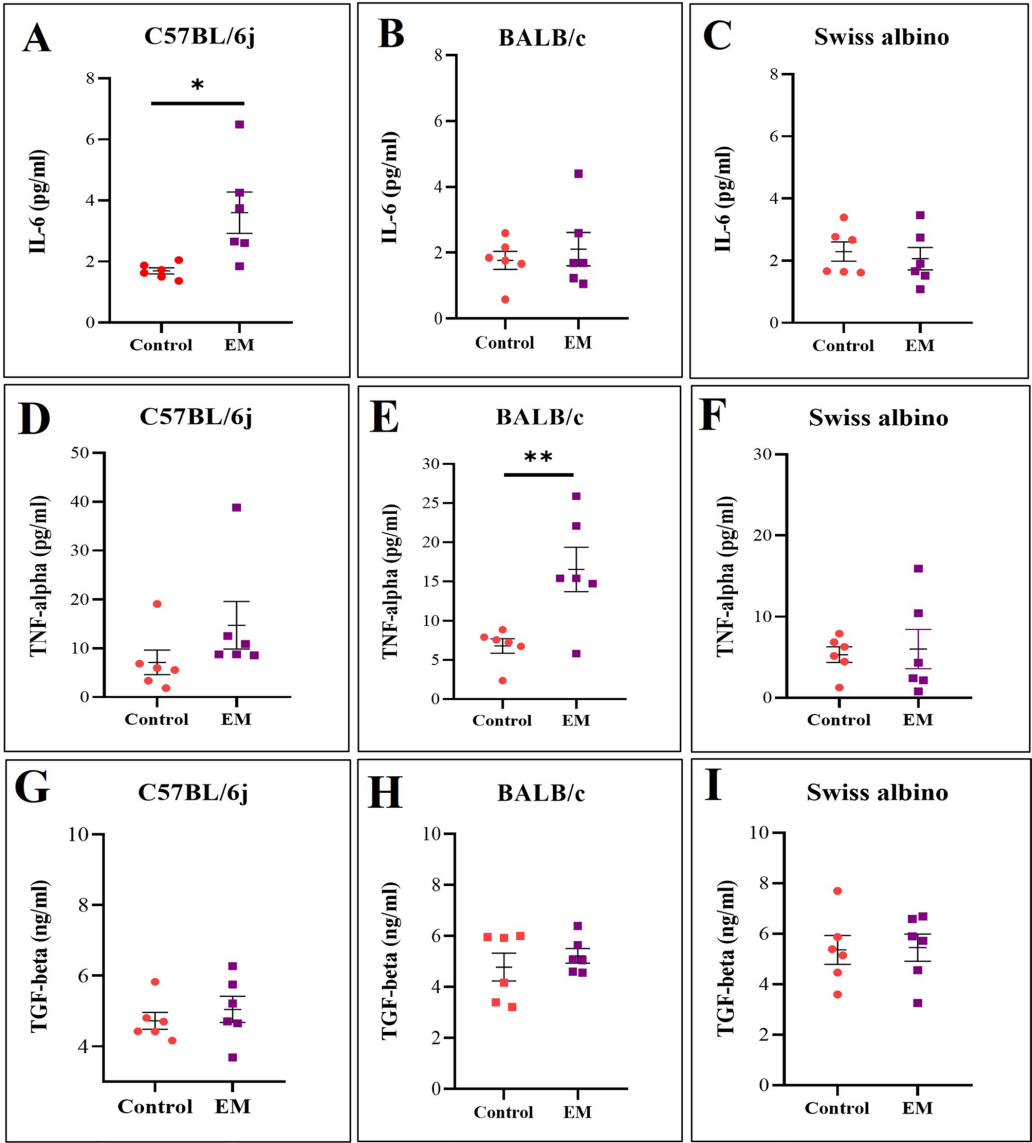
ELISA for pro-inflammatory cytokine across 3 strains. LB + mice showed enhanced pro-inflammatory cytokine expression: LB + mice showed enhanced pro-inflammatory cytokine expression: Quantification of serum cytokine levels (IL-6, TNF-α, and TGF-β) in control and EM mice across the three strains. **A** IL-6 levels were significantly increased in C57BL/6j EM mice (*p value < 0.05), and (**B**) BALB/c EM mice showed a marginal increase, while (**C**) Swiss albino EM mice displayed no variation when compared to their respective controls. **D** C57BL/6j EM mice displayed a marginal increase, while (**E**) TNF-α levels were significantly elevated in BALB/c EM mice, and (**F**) Swiss albino mice exhibited no change (**p value < 0.01) as compared to controls. **G** C57BL/6j and (**H**) BALB/c EM mice demonstrated a marginal elevation, whereas the (**I**) Swiss albino group did not show much variation as compared to the control group. Data reflect mean ± SEM and *p* value and t value generated by an unpaired t test from control *n* = 6 and EM *n* = 6 for all three strains. *p value < 0.05, **p value < 0.01 represent statistically significant values

To assess systemic neuroinflammation in EM mice, we quantified inflammatory cytokines in the blood and compared the levels to those measured at baseline in control mice. To explore the relationship between systemic cytokine expression, lesion presence, and non-evoked altered behavior, we examined cytokine profiles to burrowing performance across mouse strains. ELISA analysis of serum samples revealed strain-specific changes in inflammatory cytokine levels. IL-6 levels were significantly increased in C57BL/6j EM mice (green panel; t value = 2.786, *p value < 0.05) and showed a marginal increase in BALB/c EM mice (t value = 0.5914) with no significant difference in Swiss albino EM mice (t value = 0.4873) (Fig. 9A). TNF-α levels (blue panel) were significantly elevated in BALB/c EM mice (t value = 3.284, **p value < 0.01, with a slight increase in C57BL/6j EM mice (t value = 1.39) and no change in Swiss albino EM mice (t value = 0.2654) (Fig. 9B). TGF-β levels (orange panel) did not show significant differences among the EM (*n* = 6) and control groups (*n* = 6) for any of the three strains (C57BL/6j t value = 0.7291; BALB/c t value = 0.7214; Swiss albino t value = 0.1172) (Fig. 9C). Data reflect mean ± SEM and t value and p value generated by the unpaired t test from control *n* = 6 and EM *n* = 6 for all three strains. (*p value < 0.05, **p value < 0.01 represent statistically significant values.

## Discussion

**Fig. 10:**
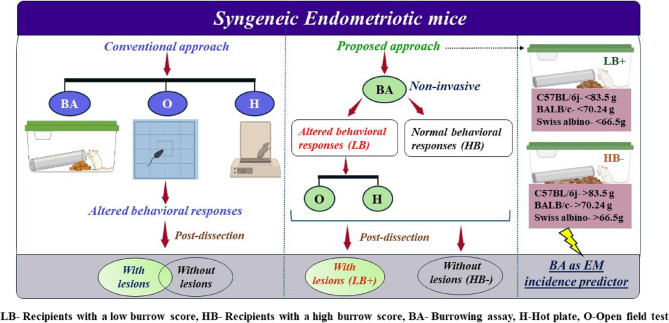
Schematic flow chart of BA for predicting EM incidence. In the proposed approach, BA is initially utilized as a non-invasive screening proxy, supported by evoked and non-evoked behavioural experiments. The burrow score cut-off differentiates low burrow score lesion-bearing EM mice (LB+) from high burrow score no-lesion-bearing EM mice (HB+), hence validating the occurrence of EM. The strain-specific cut-off values established were: C57BL/6j: <83.5 g, BALB/c: <70.24 g, and Swiss albino: <66.5 g. EM mice falling below these limits consistently showed the formation of ectopic lesions, confirming BA as an EM incidence measure in murine EM models

In recent years, a massive amount of published research has been conducted using mouse models of EM to find novel therapeutics. However, a translational gap often exists between successful preclinical treatment strategies and real-world efficacy [32]. The main cause of this discrepancy is the lack of thorough verification of EM lesion formation in preclinical studies, resulting in interventions being tested on animals that might not have even developed the condition. Moreover, EM does not occur spontaneously in rodents, likely due to the absence of endometrial shedding during the estrous cycle. To utilize this paradigm, homologous or heterologous UF must be surgically implanted or s.c/i.p injected into the peritoneal cavity of mice [33]. Regarding the preclinical models of EM, the syngeneic model is the second most common model, where the main method of delivery is intraperitoneal injection of UFs (62% of publications) without causing immunological rejection. Additionally, it does not prime the original immune system, unlike surgical administration [34]. Therefore, we chose to implement the syngeneic model of EM for our research. The inherent variability in the lesion establishment rate may be due to factors such as estrogen, the immune system, genetic background, and varied inflammatory responses [35]. In our study, we observed lesion development rates of 84.61% in C57BL/6j (*n* = 26), 84.37% in BALB/c (*n* = 32), and 67.85% in Swiss albino mice (*n* = 28), highlighting this inherent variability. The Swiss albino strain is generally known to have a different immunological profile compared to the C57BL/6j and BALB/c strains [36], which likely contributes to this reduced lesion formation success. Overall, our recent work has demonstrated that among different strains of syngeneic mice, the Swiss albino strain fared poorly in terms of phenotypes of fibrosis and pain-associated symptoms [26]. The substantially reduced lesion incidence in Swiss albino mice accounts for their behavioral profile differences across behavioral experiments. This variation emphasizes the importance of identifying precise behavioral markers of lesion presence, since not all EM-induced animals developed ectopic lesions (Fig. 10).

Moreover, there are inconsistencies in how disease burden is captured in animal models [37]. The behavioral assays in animal models rely primarily on evoked responses to external stimuli, whereas clinical evaluations emphasize spontaneous and ongoing symptoms reported by patients [38]. Existing non-surgical EM models often fail to incorporate reliable behavioral markers to evaluate ongoing spontaneous pain and lesion incidence. This created a significant gap in replicating patient symptomatology in vivo [39]. Thus, there is a pressing need for a murine model that can closely mirror the clinical heterogeneity of EM by integrating lesion incidence with behavioral readouts, thereby bridging the gap between animal and clinical research.

One of the continuing obstacles in preclinical EM research is the lack of a 100% induction success rate. One of the primary reasons for the discrepancy is that EM mice used in pre-clinical research often with unconfirmed lesion formation. The available induction procedures, such as surgical implantation or intraperitoneal injection of UF, failed to ensure consistent lesion establishment since the lesion formation is influenced by various other factors, such as estrogen, the immune system, genetic background, and varied inflammatory responses [40]. Several studies show promising effects in lesion regression or pain reduction without accounting for whether all experimental animals effectively developed EM before intervention. This diversity hampers the evaluation of therapy outcomes, as it remains unclear whether the absence of lesions at the study endpoint signifies the treatment efficacy or merely unsuccessful model induction [6–9]. These limitations highlight the necessity for fully verified EM models that provide consistent evaluation of lesion existence before and post treatment. The correlation between pain severity and EM presentations remains inadequately defined [39]. However, epidemiological evidence suggests that the relationship between pelvic pain intensity and EM is independent of the lesions’ size, anatomical locations, or number of lesions and EM stage [41, 42]. Additionally, the sole approach to validate EM formation is through endpoint dissection, which poses a considerable obstacle for translating findings to clinical therapeutic applications. While EM pain may be provoked by physical stimuli (e.g., dysuria, dyspareunia, dyschezia), a significant portion of the chronic pain lacks a definitive mechanical trigger [43]. Thus, measuring non-evoked behavioral responses in mouse models may better reflect patient experiences and facilitate translational studies. Hence, we wanted to study whether altered burrowing behavior can be a biomarker of EM incidence. Then we investigated pain or general discomfort using both spontaneous and evoked behaviors. Burrowing, one of the social habits of rodents, is significantly conserved in laboratory rodents [19]. Bashir et al. have recently demonstrated that EM mice showed reduced burrowing compared to control animals [25]. Their findings align with our hypothesis, and accordingly, we observed a significant and consistent decrease in burrowing performance of EM mice compared to the control mice. This demonstrates traits similar to those of symptomatic women with EM, who reported a diminished quality of life and heightened psychological distress. The behavioural shift was also complied with the presence of EM lesions post-sacrifice. The combined analysis of burrowing data from all three strains using ROC curve analysis yielded a combined AUC of 0.883 (model quality 0.95), demonstrating that a BA is a highly sensitive test for the presence of lesions within this experimental context. We observed a weak correlation between burrow score and lesion number(r^2^ = 0.008). This lack of a strong correlation closely mirrors the clinical presentation of EM in human patients. In women, the severity of chronic pelvic pain is often independent of the lesion size, anatomical location, or the number of lesions. Thus, BA effectively predicts the presence of the disease and associated discomfort, but the resulting low burrowing is not solely governed by the quantity of the ectopic lesions.

A drawback of most previous investigations is the absence of additional validation to verify decreased burrowing activity as a pain-specific outcome. So, it is essential to compare non-evoked burrowing behaviour-related outcomes with other pain metrics to better understand their interrelation. For instance, in a study examining neuropathic pain in rats, burrowing performance exhibited no correlation with evoked mechanosensory thresholds, concluding decreased burrowing may signify elements of pain, which are not directly associated with increased sensory responses [44]. This constraint leaves the relationship between burrowing behavior and pain largely unknown, encouraging further investigation. Given that EM frequently causes significant pelvic pain that affects both the evoked and non-evoked responses, this finding has clinical significance [45]. Also, it has been hypothesized that the pain reported in burrowing tests is spontaneous, rather than evoked pain, as earlier studies revealed no association between the quantity of material burrowed and an evoked paw withdrawal measurement [44, 46]. In contrast, we showed a correlation between lower burrowing activity and pain-response assays. Hence, these correlations enhance the utility of burrowing as a behavioral predictor of pain and disease incidence [47]. To our knowledge, this is the first study to show combined evoked and non-evoked pain responses and their correlation with burrowing behavior in a syngeneic mouse model of EM. Compared to the control mice, which showed a distinctly disrupted, scattered exploring pattern, characterized by an increase in the number of central entries, the EM mice exhibited decreased exploratory behavior and increased anxiety-like behavior, spending more time in the peripheral zones [27]. EM mice also exhibited hyperalgesia. Given that EM frequently causes significant pelvic pain that affects both evoked and non-evoked responses, this finding has clinical significance [45]. It has been previously hypothesized that the pain reported in BA is more akin to spontaneous rather than evoked [44, 46]. However, our findings reveal a clear correlation between poor burrowing activity and heightened sensitivity in evoked behavioral assays, further strengthening the validity of burrowing as a behavioral predictor of lesion incidence in this context [47].

Our preclinical mouse model also confirms its significance by assessing the levels of a subset of pro-inflammatory markers in the serum of EM mice to determine the presence of a local inflammatory milieu (51). Previous findings demonstrated that local inflammation is a crucial mediator in adhesion formation, with IL-β, IL-6, and TNF-α identified among major participants [48, 49]. TNF-α has been involved in early phases of EM progression in mice, and its rise has been linked with chronic pelvic pain [50–52]. We discovered increased pro-inflammatory cytokines (IL-6, TNF-α, and TGF-β) in the serum of C57BL/6j and BALB/c EM mice, but not in Swiss albino mice. This strain-specific variance underlines the heterogeneity for strain-specific changes in cytokine responses. The elevated cytokine levels we observed presumably contribute to both lesion persistence and pain onset.

### Limitation

We acknowledge that this study has several limitations. The absence of a non-invasive imaging tool to longitudinally validate BA findings and directly correlate burrowing behavior with lesion development and progression represents a potential area for improvement. The absence of a validated observational tool, such as the Mouse Grimace Scale (MGS), is another limitation of our work. MGS reflects an animal’s spontaneous, ongoing discomfort rather than a reflexive withdrawal from an external stimulus, as seen in the Hot Plate assay, or general anxiety as seen in the OFT. Therefore, its inclusion would have provided a more direct and robust measure of the chronic pain associated with endometriosis. Similarly, using some analgesic interventions to assess the sensitivity of the BA to relieve pain would further strengthen its utility as a biomarker. We did not delve into the underlying mechanisms responsible for this altered behavior in the presence of ectopic lesions. Furthermore, we could only validate BA via a classical syngeneic mouse model. Further validation using alternative model-generation methods will be necessary to make informed decisions about the universal applicability of this biomarker. We acknowledge a limitation regarding the small sample size of EM mice that did not develop ectopic lesions (HB- mice). Across all strains, only 20 recipient mice (C57BL/6j = 6, BALB/c = 5, Swiss albino = 9) fell into the HB- group, which is a consequence of the relatively high lesion induction rate observed (up to 84.61%). This low n value for HB- mice, especially in the BALB/c and Swiss albino behavioral sub-cohorts (*n* = 4), inherently reduces the statistical power of the comparisons between HB- and control mice. Finally, while we defined a cut-off value for poor burrowing behavior based on ROC analysis, a more detailed exploration of the criteria used to classify mice for subsequent pain assays could enhance the transparency of our methodology. Although the BA proved highly effective as a lesion-presence predictor (AUC = 0.981), the possibility of lesion-positive animals that exhibit normal burrowing behavior (false negatives) exists and should be investigated in future studies. Nevertheless, our current findings strongly validate the BA score as a simple and effective measure to select lesion-positive animals with greater confidence. The low number of HB- animals means the sample size for investigating potential false negatives is limited. Therefore, while our data strongly support the BA’s utility, future multicentric studies involving significantly larger cohorts must investigate the false-negative rate and replicate these HB- behavioral findings to solidify the BA’s universal applicability and translational utility. Additionally, although our current data aligns with the clinical paradox that pain severity is often independent of lesion number, future investigations should delve into the underlying mechanisms that govern the lack of correlation between ectopic lesion load and the magnitude of spontaneous burrowing behavior.

## Conclusion

Our research demonstrates the feasibility of utilizing an inexpensive burrow tube as a cost-effective, non-invasive instrument for assessing lesion burden in EM mice before endpoint analysis. In conclusion, when used as a biomarker, classical burrowing behavior serves as a reliable biomarker, yielding a high-confidence assessment of EM incidence. This finding underscores the potential of BA as a vital tool in designing and evaluating preclinical studies, facilitating the distinction between animals with lesions and those without.

## Supplementary Information

Supplementary Material 1.

## Data Availability

The original contributions presented in the current study are included in the article; Further inquiries can be directed to the corresponding author.

## Abbreviations

EM: Endometriosis
ROC: Receiver Operating Characteristic
i.p.: Intraperitoneal
UF: Uterine Fragments
BA: Burrowing Assay
LB+: Low burrow score and lesion positive
HB: -High burrow score and lesion-negative
OFT: Open Field Test
µL: microliter
PF: Peritoneal Fluid
PBS: Phosphate-Buffered Saline
DAB: Diaminobenzidine
BSA: Bovine Serum Albumin
HRP: Horseradish Peroxidase
mg/kg: Milligram per kilogram
pg/mL: Picogram per milliliter
SEM: Standard Error of Mean
ANOVA: Analysis of Variance
LR: Linear Regression
SPSS: Statistical Package for the Social Sciences
