# Supplementary material for: Burrowing behavior is a potential non-invasive proxy for lesion development in a syngeneic murine model of endometriosis
Source: BMC Womens Health. 2025 Dec 12;26:39. doi: 10.1186/s12905-025-04112-4 (PMC12822023; doi:10.1186/s12905-025-04112-4)
Supplement: Supplementary file 1 — Supplementary Material 1. [file 12905_2025_4112_MOESM1_ESM.docx]

# Table 1 defines supplementary OFT parameters in control and LB mice among all three strains, in addition to alterations in the number of central entries, time spent in the center, and peripheral zones.

| **Table 2** | **C57BL/6j** | | **BALB/c** | | **Swiss albino** | |
| --- | --- | --- | --- | --- | --- | --- |
|  | **Control** | **LB** | **Control** | **LB** | **Control** | **LB** |
| **Central entries (n)** | 36.25±12.  24 | 19±9.133** | 34.25±7.3  4 | 12.37±6.26*  *** | 37.25±16.  04 | 29.25±15.0  7 |
| **Central time (s)** | 39.13±21.  70 | 17.5125±11.  385** | 69.27±49.  96 | 26.41±8.92* | 35.43±18.  23 | 26.32±11.4  2 |
| **Peripheral time (s)** | 554.28±7  2.07 | 618.693±90.  215 | 498.40±9  5.67 | 611.10±69.8  2* | 569.66±1  04.06 | 677.39±66.  53^*^ |
| **Total distance**  **travelled (m)** | 55.45±12.  01 | 42.43±17.56  1 | 59.43±10.  41 | 32.83±9.71*  ** | 43.52±11.  98 | 36.41±6.40 |
| **Mean speed (m/s)** | 0.06±0.01  3 | 0.047±0.019 | 0.06±0.01  1 | 0.03±0.008*  *** | 0.04±0.01  4 | 0.04±0.007 |
| **Freezing episodes**  **(n)** | 1.87±1.05 | 7.125±8.0 | 0.75±0.82 | 4.5±3.64* | 0.6±0.99 | 2.75±4.43 |
| **Freezing time (s)** | 7.03±4.97 | 26.35±27.73 | 2.35±2.71 | 10.57±8.63* | 3.57±6.85 | 9.81±15.81 |
| **Total mobile time**  **(s)** | 719.57±6  5.27 | 544.8±126.3  7** | 789.97±2  7.54 | 663.10±69.2  2*** | 700.91±7  8.86 | 612.64±104  .36 |
| **Total immobile**  **time (s)** | 180.42±6  5.27 | 355.2±126.3  7** | 92.94±25.  35 | 230.35±73.7  5 | 195.6±77.  54 | 283.87±106  .31 |

# Table 2 defines supplementary OFT parameters in control and HB mice among all three strains, in addition to alterations in the number of central entries, time spent in the center, and peripheral zones.

#

| **Table 3** | **C57BL/6j** | | **BALB/c** | | **Swiss albino** | |
| --- | --- | --- | --- | --- | --- | --- |
|  | **Control** | **HB** | **Control** | **HB** | **Control** | **HB** |
| **Central entries (n)** | 36.25±12.  24 | 24.87±6.39* | 34.25±7.3  4 | 40.25±6.94 | 37.25±16.  04 | 39.25±13.32 |
| **Central time (s)** | 39.13±21.  70 | 24.32±10.71 | 69.27±49.  96 | 39.25±13.32 | 35.43±18.  23 | 39.45±19.79 |
| **Peripheral time (s)** | 557.9 ±64.19 | 543.07±120.81 | 498.40±9  5.67 | 688.507±74** | 569.66±1  04.06 | 632.76±56.36 |
| **Total distance**  **travelled (m)** | 55.45±12.  01 | 53.71±16.33 | 59.43±10.  41 | 64.68±8.43 | 43.52±11.  98 | 41.28±21.92 |
| **Mean speed (m/s)** | 0.06±0.01  3 | 0.05±0.017 | 0.06±0.01  1 | 0.07±0.009 | 0.04±0.01  4 | 0.06±0.008** |
| **Freezing episodes**  **(n)** | 1.87±1.05 | 1.75±0.96 | 0.75±0.82 | 1.5±1.5 | 0.6±0.99 | 0.25±0.43 |
| **Freezing time (s)** | 7.03±4.97 | 9.5±12.05 | 2.35±2.71 | 5.6±5.94 | 3.57±6.85 | 2.85±3.82 |
| **Total mobile time**  **(s)** | 719.57±6  5.27 | 606.46±95.35* | 789.97±2  7.54 | 738.45±3.63** | 700.91±7  8.86 | 717.46±103.79 |
| **Total immobile**  **time (s)** | 180.42±6  5.27 | 293.53±95.35* | 92.94±25.  35 | 111.6±96 | 195.6±77.  54 | 188.05±71.7 |

#

**Fig. S1:**

**Fig. S1:** Cumulative mean (±SEM) body weight vs. time for the control and the EM mice after i.p injection of UF or 1x PBS. There were no significant (P > 0.05) differences in the mean (±SEM) body weights throughout the experimental period when compared with the respective control mice, indicating overall health remained uncompromised, confirming the UF injections did not have any negative impact on recipients.


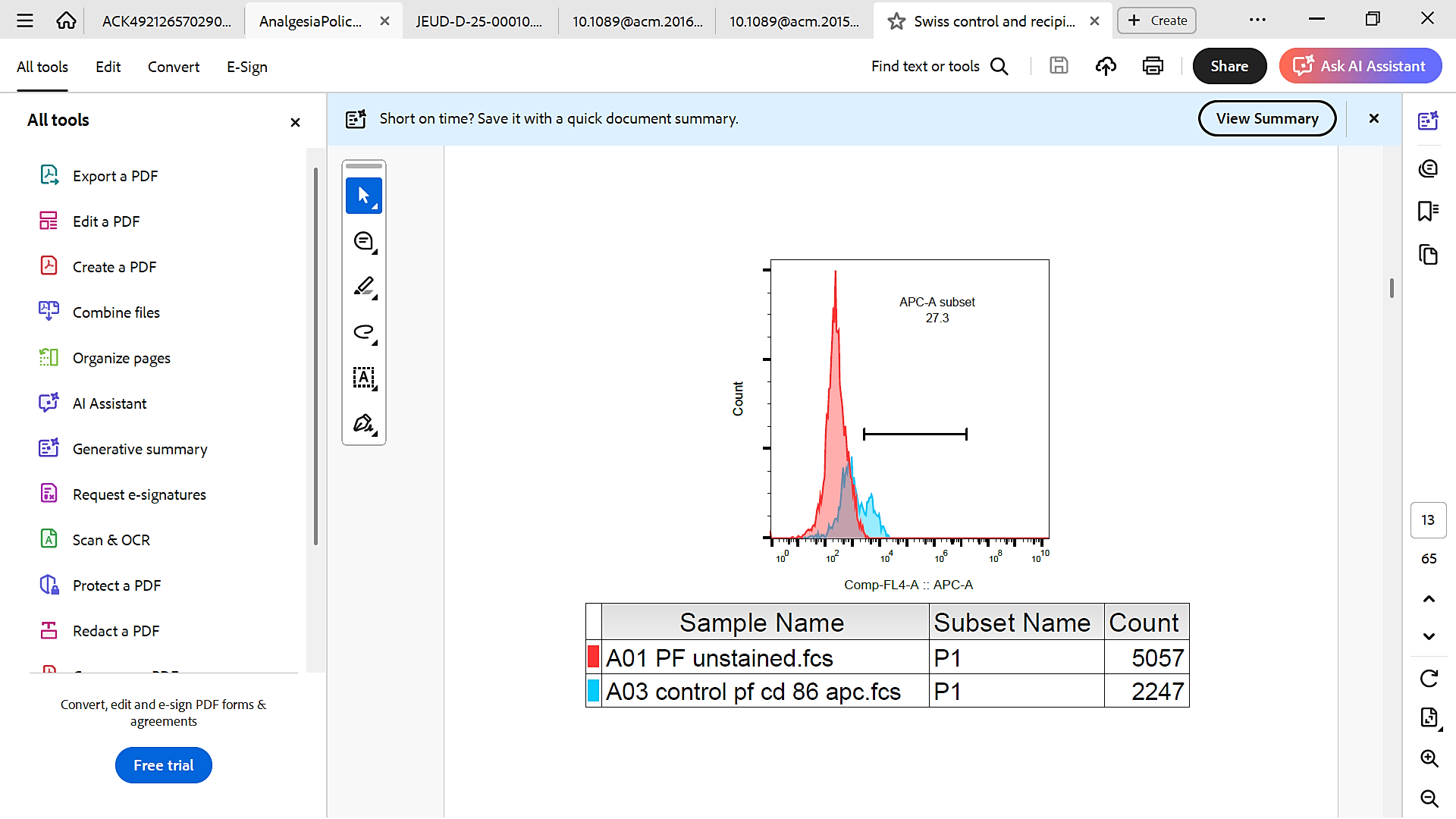


**Swiss albino BALB/c C57BL/6j**

**CD86 (M1) CD206 (M2)**

Control EM Control EM


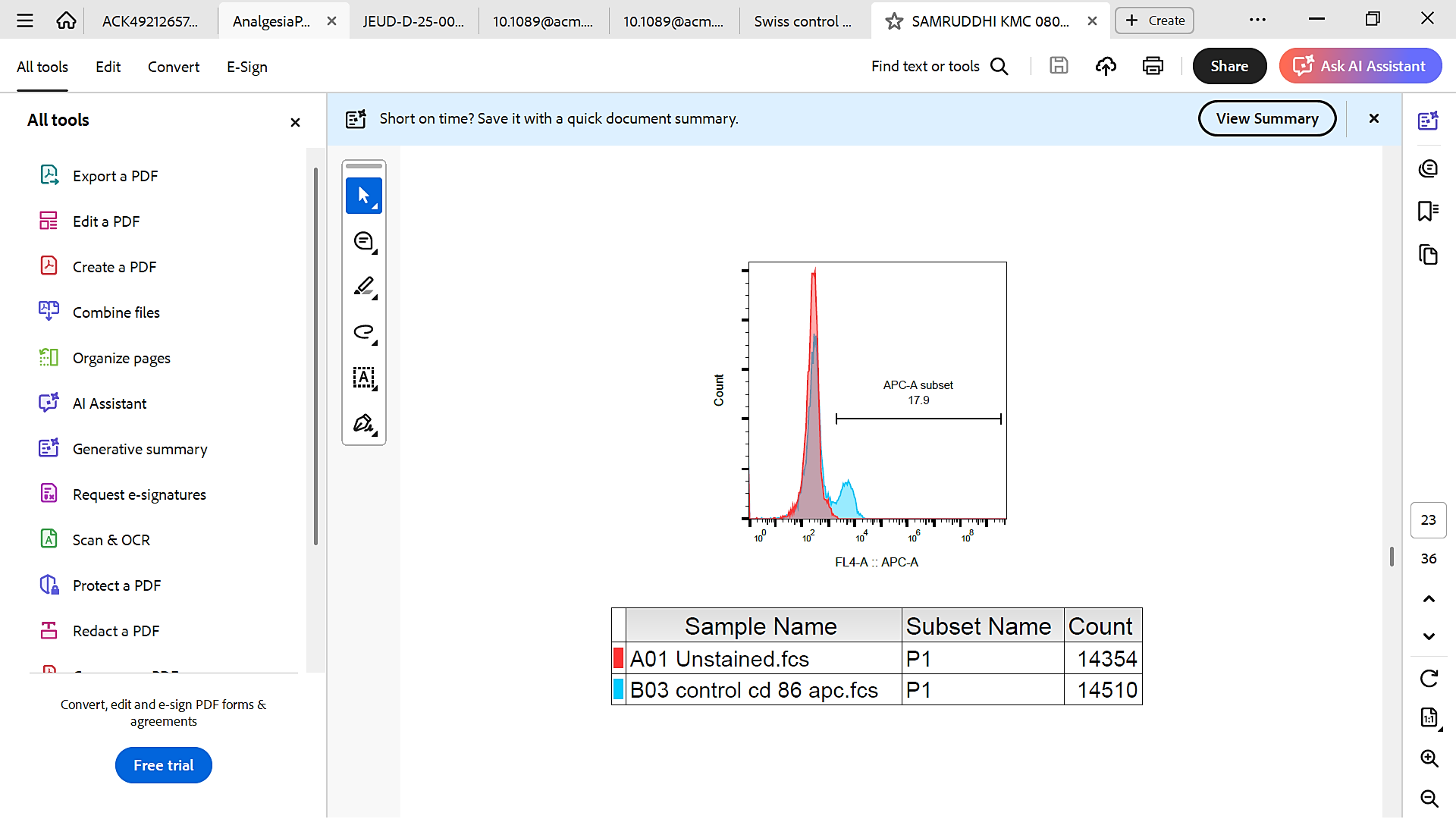

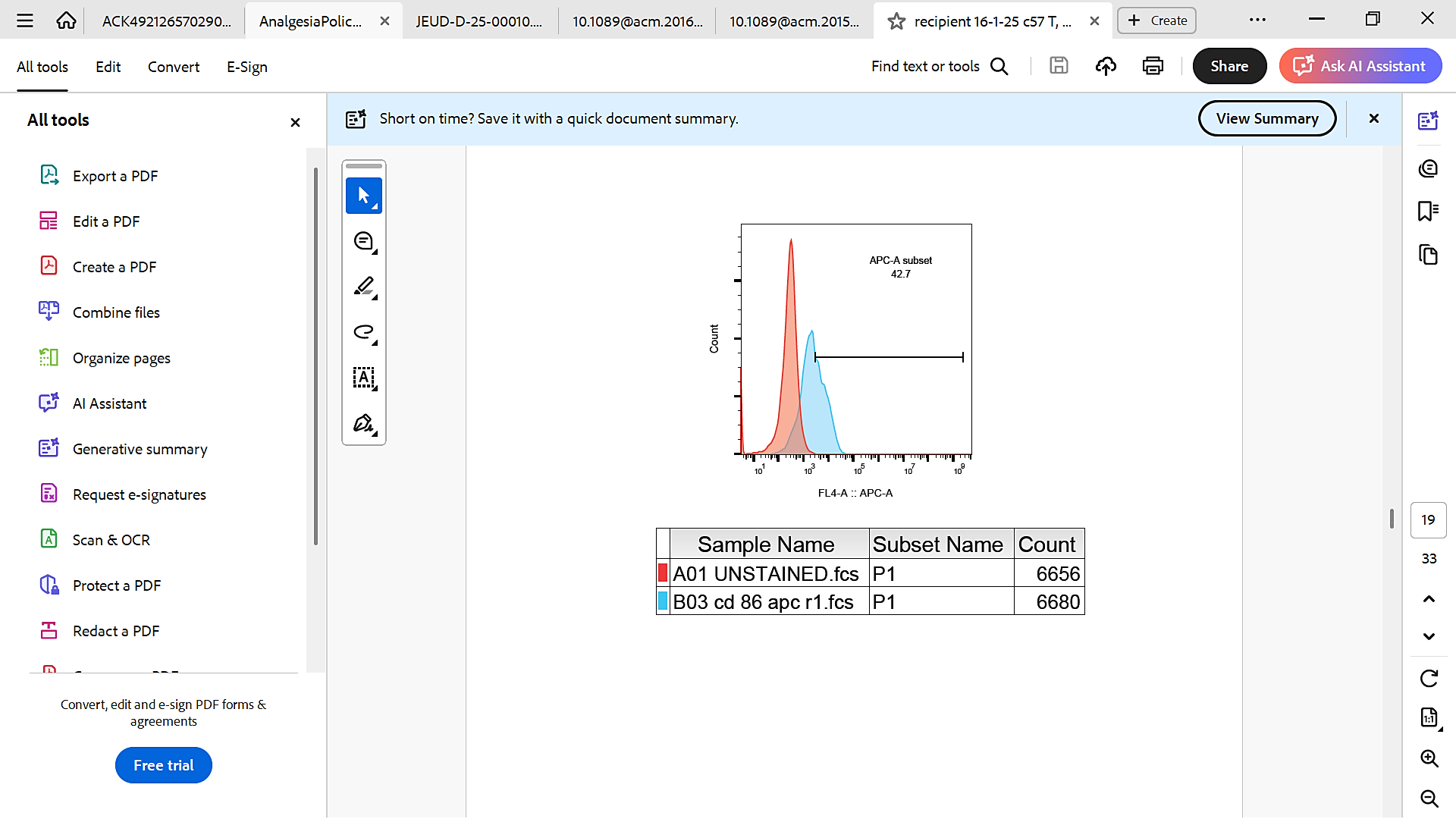

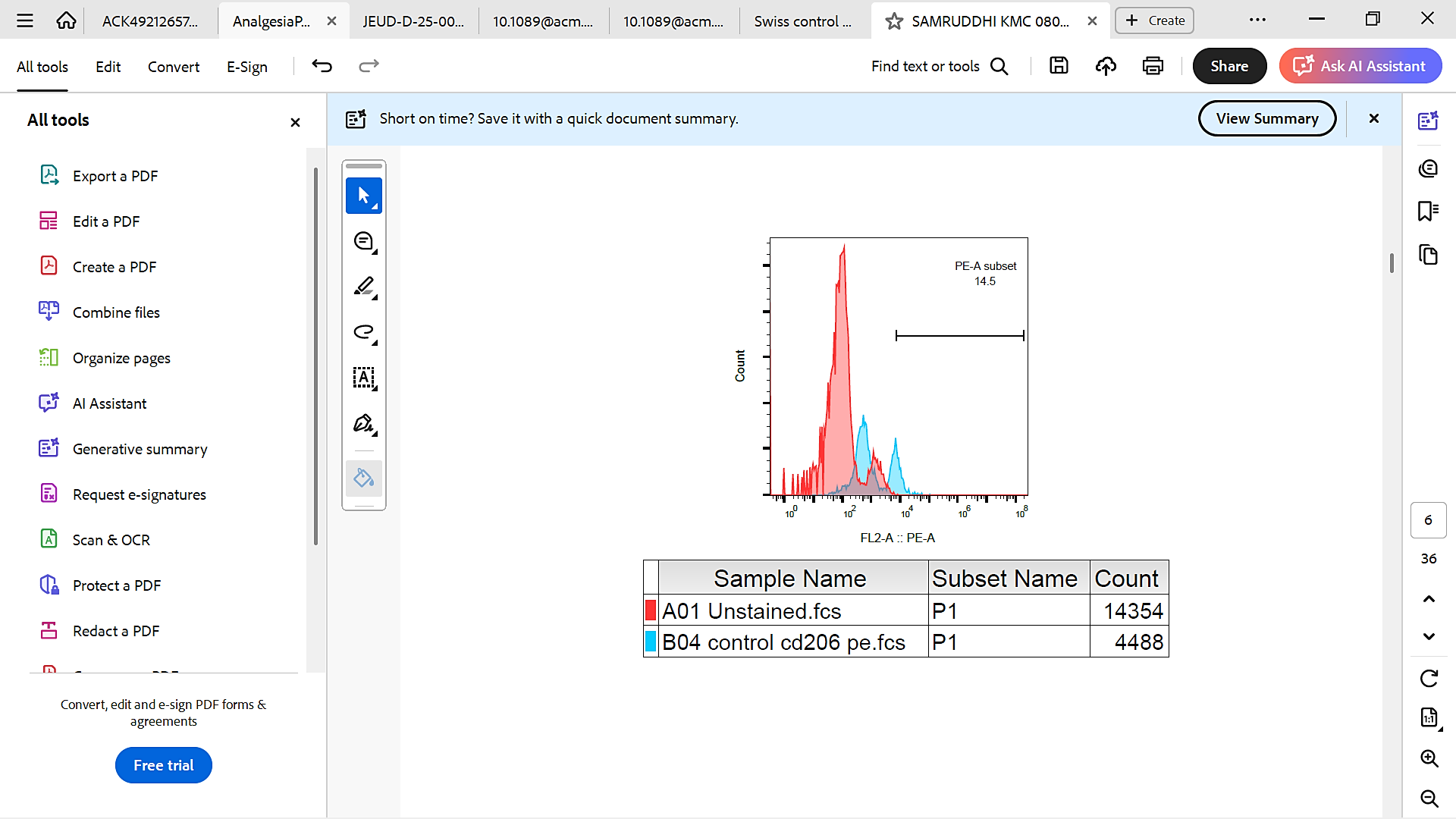

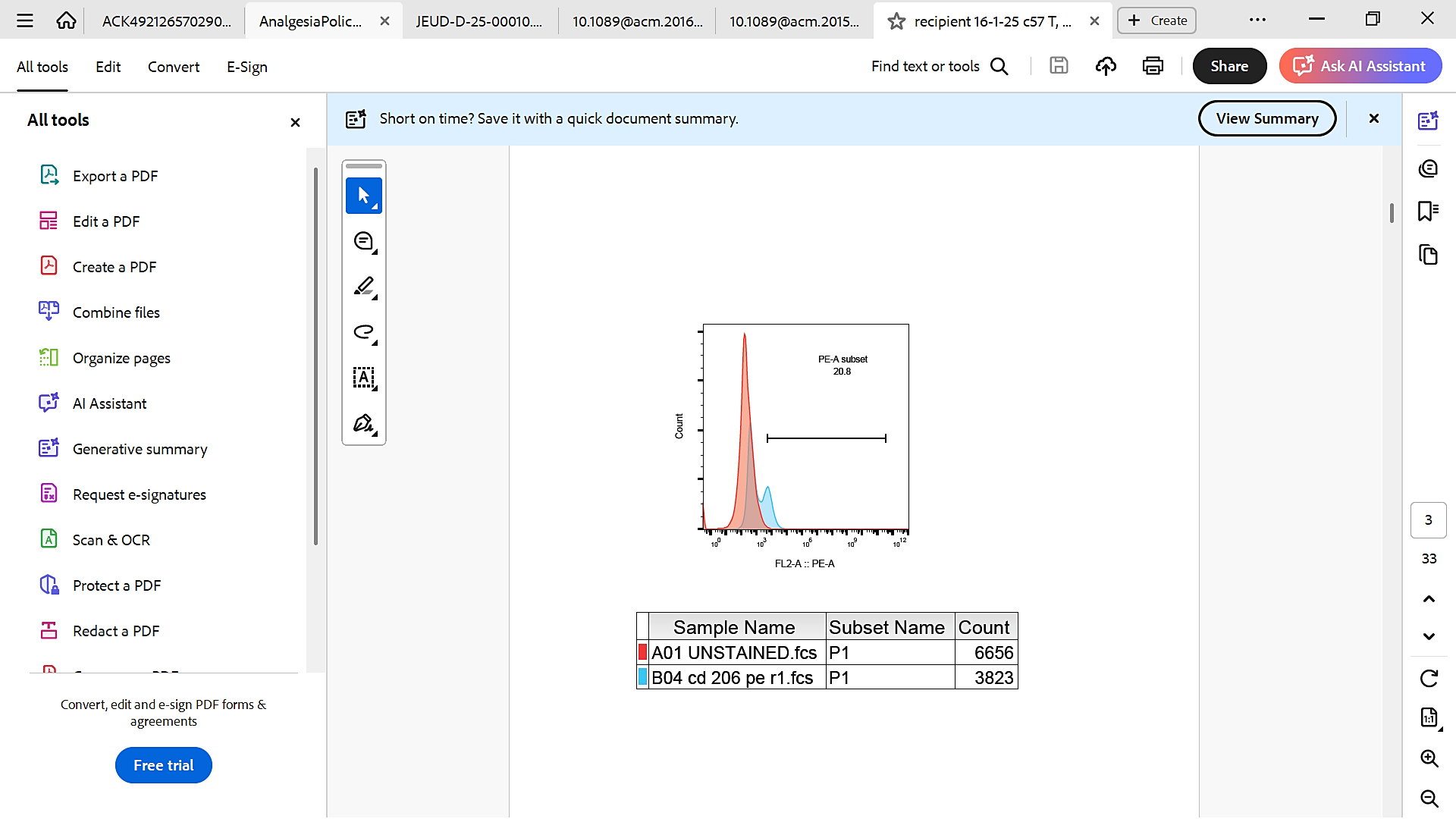

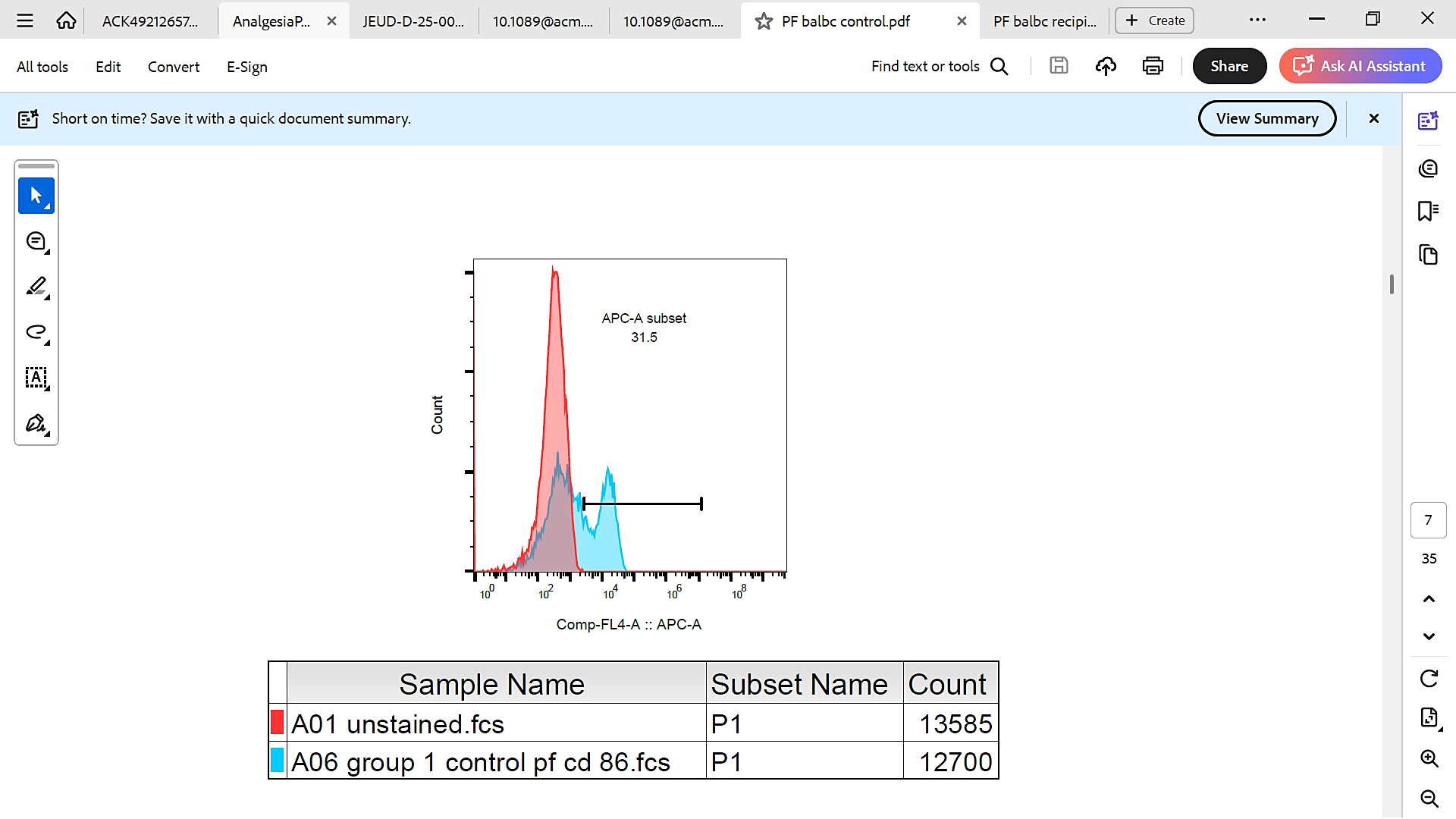

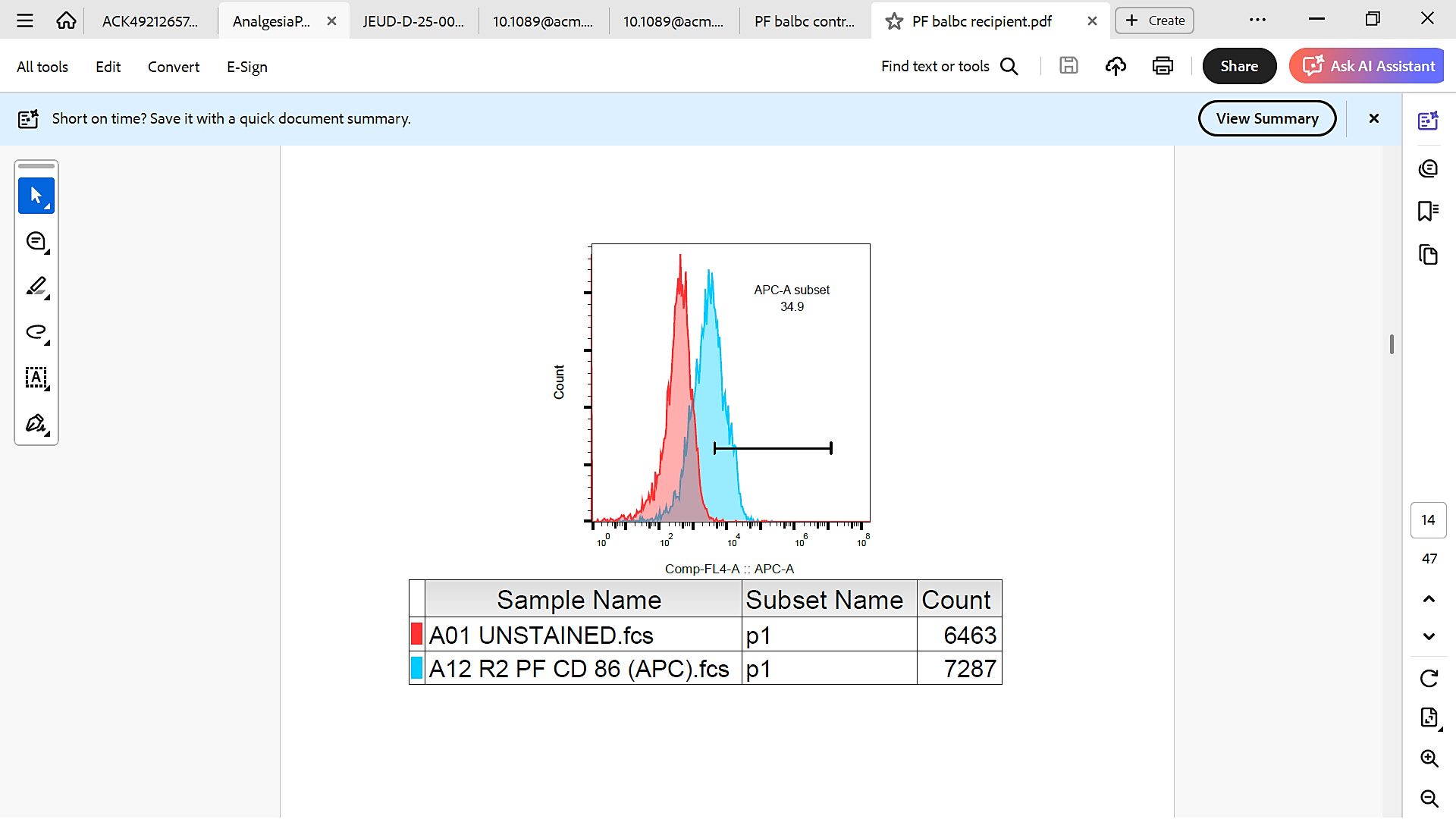

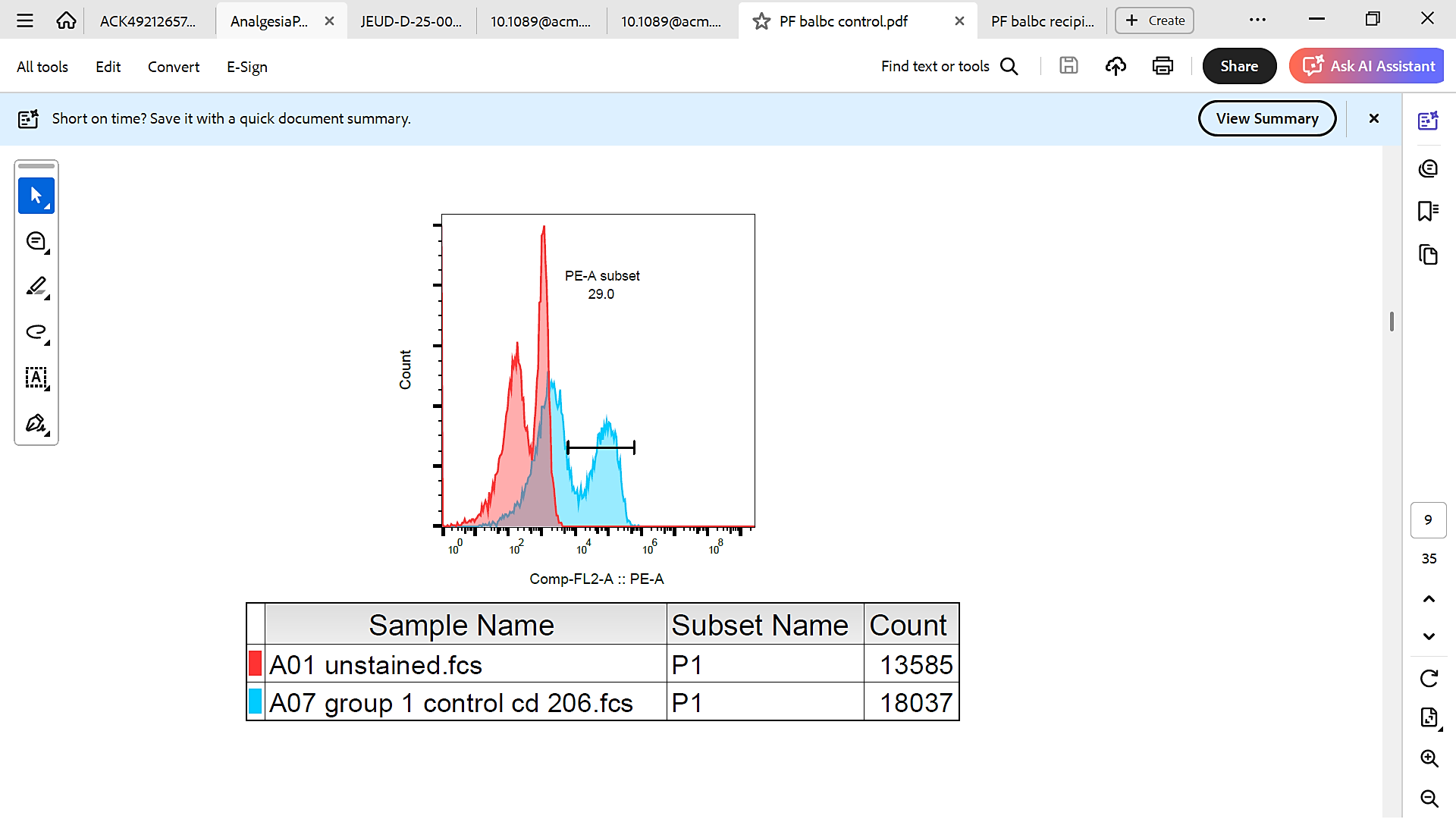

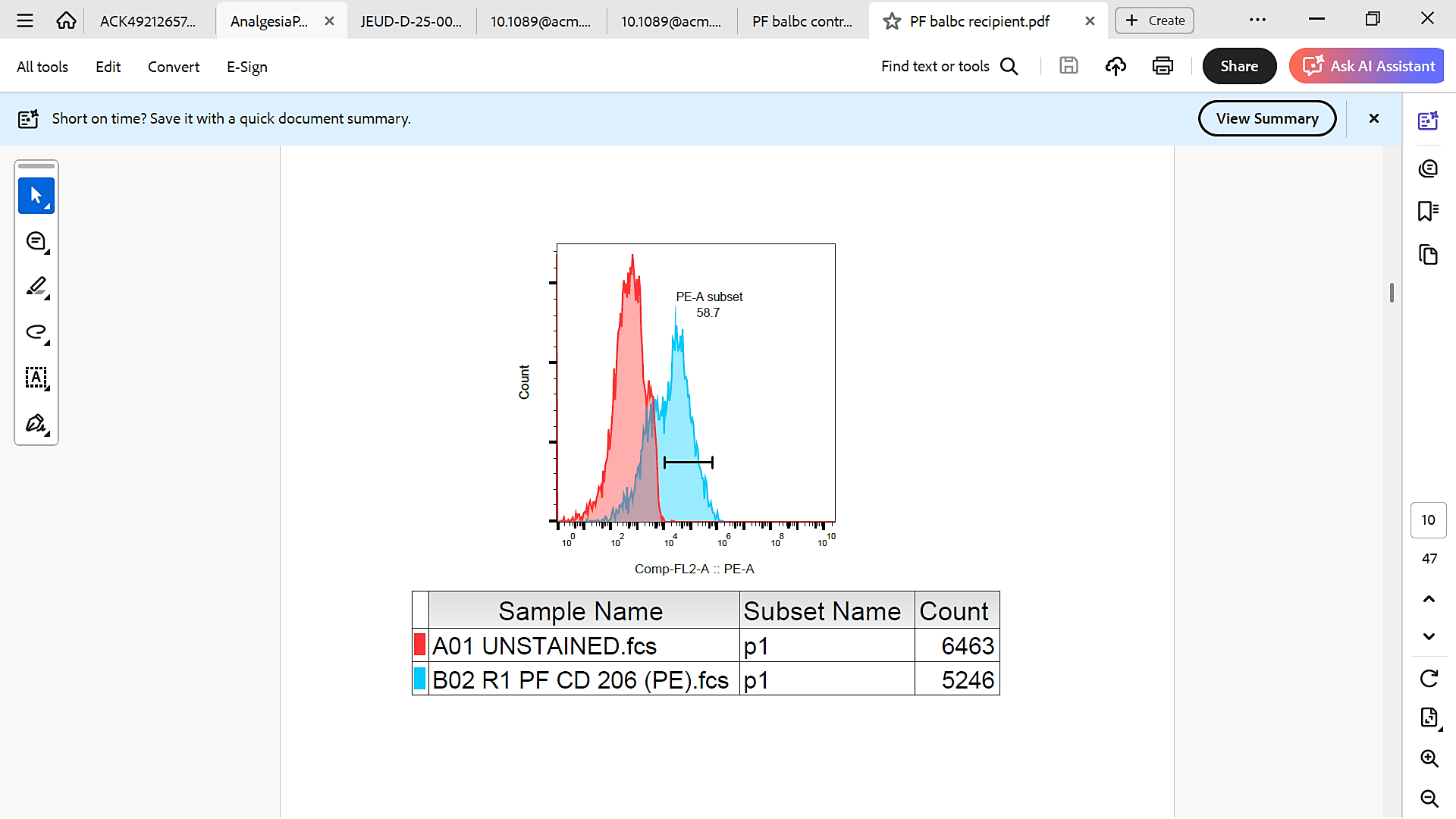

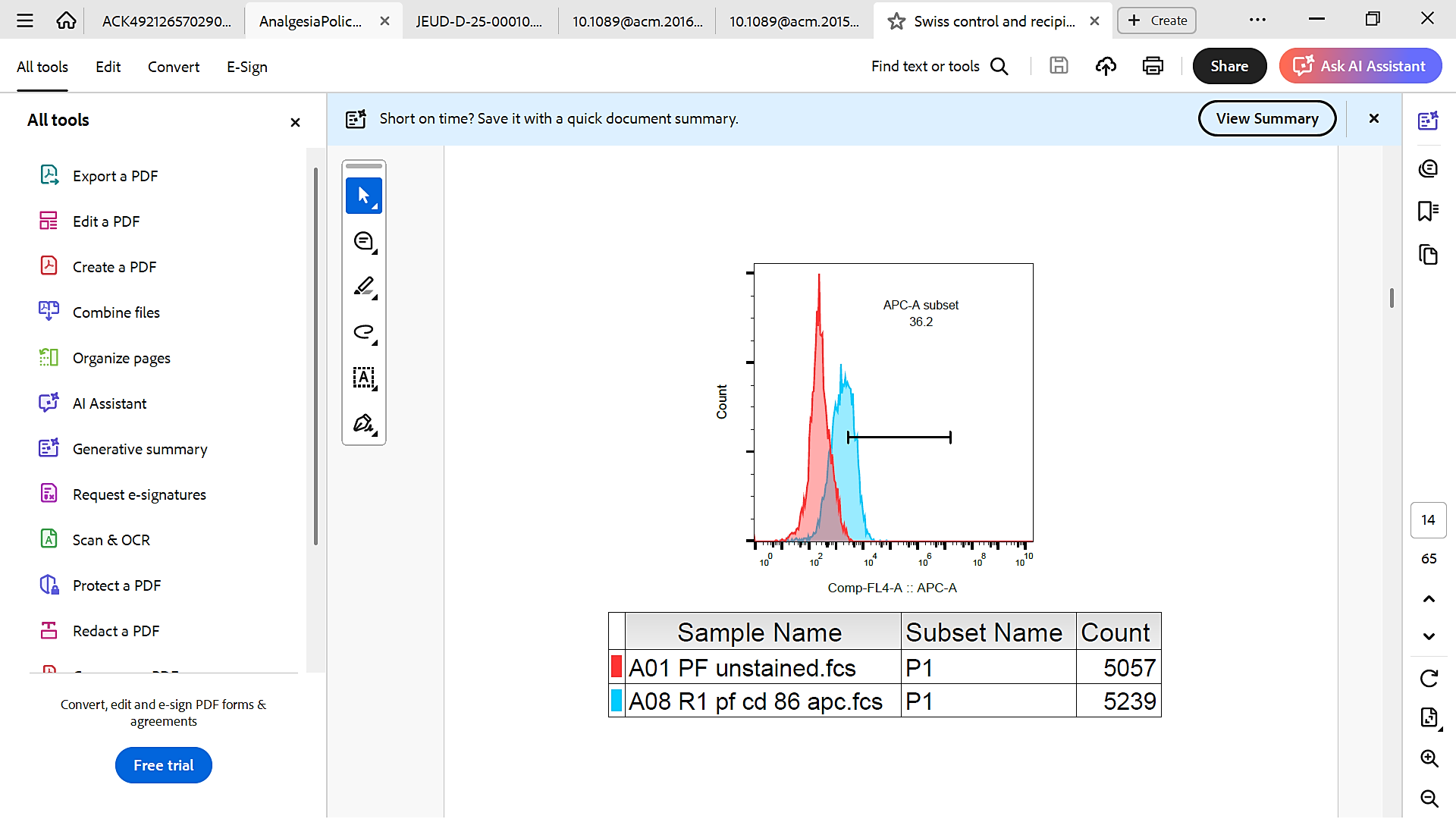

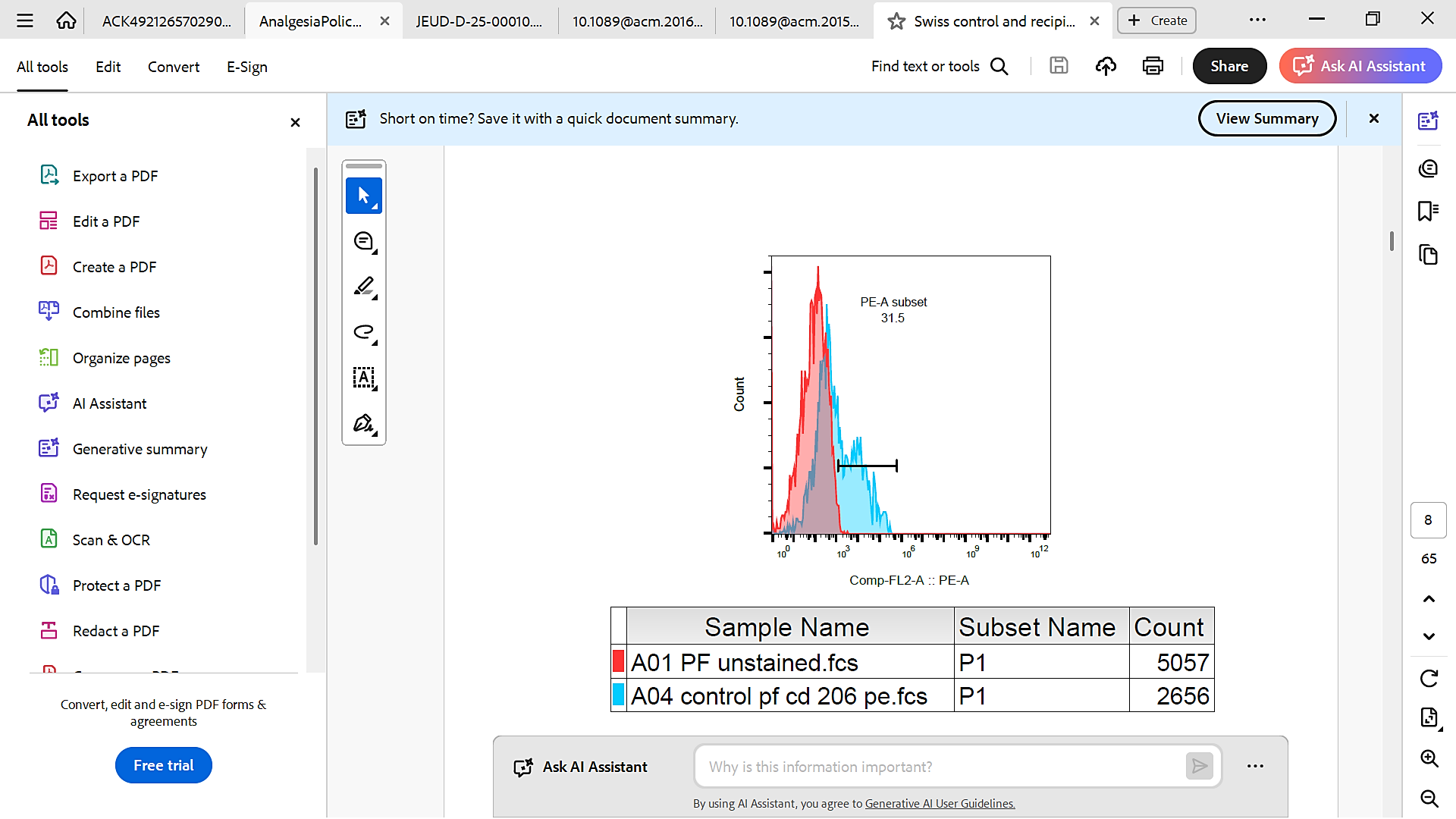

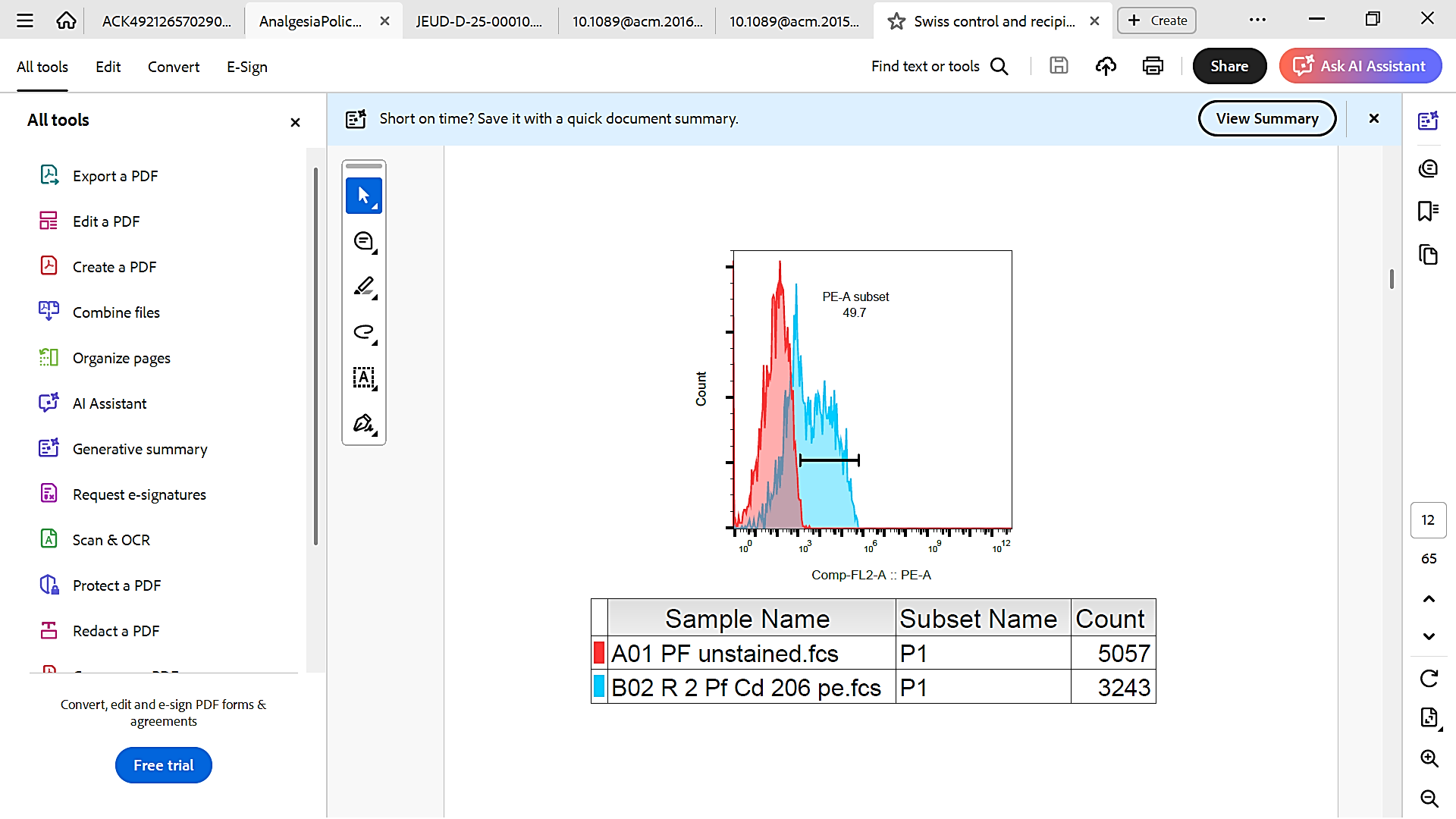


**Fig S2:** FC Analysis of peritoneal macrophage subpopulations in EM mice. PF was collected from control and EM mice of three strains (C57BL/6j, BALB/c, and Swiss albino) and analyzed by FC to assess M1 and M2 macrophage populations. Live, single cells were gated for analysis. (a) M1 macrophage population: The percentage of M1 macrophages, identified as CD11+CD86+ cells, showed a marginal increase in EM mice compared to controls for C57BL/6j (26.5% vs. 12.8%), BALB/c (24.9% vs. 20.1%), and Swiss albino (20.7% vs. 17.2%). (b) M2 Macrophage population: The percentage of M2 macrophages, identified as CD11+CD206+ cells, was significantly increased in EM mice compared to controls for C57BL/6j (27.2% vs. 10.2%), BALB/c (26.9% vs. 22.5%), and Swiss albino (25.3% vs. 10.1%).
